# Anopheles gambiae Immune Responses to Human and Rodent *Plasmodium* Parasite Species

**DOI:** 10.1371/journal.ppat.0020052

**Published:** 2006-06-09

**Authors:** Yuemei Dong, Ruth Aguilar, Zhiyong Xi, Emma Warr, Emmanuel Mongin, George Dimopoulos

**Affiliations:** 1 W. Harry Feinstone Department of Molecular Microbiology and Immunology, Bloomberg School of Public Health, Johns Hopkins University, Baltimore, Maryland, United States; 2 Department of Human Genetics and Genome Quebec Innovation Centre, McGill University, Montreal, Quebec, Canada; 3 European Molecular Biology Laboratory, European Bioinformatics Institute, The Wellcome Trust Genome Campus, Hinxton, Cambridge, United Kingdom; National Institutes of Health, United States of America

## Abstract

Transmission of malaria is dependent on the successful completion of the *Plasmodium* lifecycle in the *Anopheles* vector. Major obstacles are encountered in the midgut tissue, where most parasites are killed by the mosquito's immune system. In the present study, DNA microarray analyses have been used to compare Anopheles gambiae responses to invasion of the midgut epithelium by the ookinete stage of the human pathogen Plasmodium falciparum and the rodent experimental model pathogen *P. berghei.* Invasion by P. berghei had a more profound impact on the mosquito transcriptome, including a variety of functional gene classes, while P. falciparum elicited a broader immune response at the gene transcript level. Ingestion of human malaria-infected blood lacking invasive ookinetes also induced a variety of immune genes, including several anti-*Plasmodium* factors. Twelve selected genes were assessed for effect on infection with both parasite species and bacteria using RNAi gene silencing assays, and seven of these genes were found to influence mosquito resistance to both parasite species. An MD2-like receptor, AgMDL1, and an immunolectin, FBN39, showed specificity in regulating only resistance to *P. falciparum,* while the antimicrobial peptide gambicin and a novel putative short secreted peptide, IRSP5, were more specific for defense against the rodent parasite *P. berghei.* While all the genes that affected *Plasmodium* development also influenced mosquito resistance to bacterial infection, four of the antimicrobial genes had no effect on *Plasmodium* development. Our study shows that the impact of P. falciparum and P. berghei infection on A. gambiae biology at the gene transcript level is quite diverse, and the defense against the two *Plasmodium* species is mediated by antimicrobial factors with both universal and *Plasmodium*-species specific activities. Furthermore, our data indicate that the mosquito is capable of sensing infected blood constituents in the absence of invading ookinetes, thereby inducing anti-*Plasmodium* immune responses.

## Synopsis

The malarial parasite *Plasmodium* has to traverse the gut wall of the *Anopheles* mosquito in order to complete its lifecycle and to be transmitted between hosts. At the midgut stage of infection, the mosquito activates immune responses to eliminate most invading parasites. The features of these immune responses are not very well understood and have mainly been examined using the rodent parasite model *P. berghei.* Here the authors investigated the relationship between the Anopheles gambiae responses against the human pathogen *P. falciparum,* the rodent parasite *P. berghei,* and bacterial infections, at both the gene expression and functional levels. The mosquito responses against these pathogens were quite diverse, and the defense against the two malaria parasite species involved both common and species-specific components. Malaria-infected blood was sufficient to activate anti-*Plasmodium* immune responses, even in the absence of midgut invasion. Through this mechanism, the mosquito can initiate its defense against *Plasmodium* prior to invasion of the gut. Mosquito genes that could negatively influence *Plasmodium* development were also capable of regulating the resistance to bacterial infection, but several of the antibacterial genes had no effect on *Plasmodium;* thus, the mosquito apparently utilizes its antibacterial defense systems against the malaria parasite.

## Introduction

The transmission of the malarial parasite *Plasmodium* by the vector mosquito Anopheles gambiae is enabled by hematophagy, which is essential for egg production. Within 24 h after ingestion of infected blood, the *Plasmodium* gametocytes are fertilized and develop into motile ookinetes, which invade and traverse the mosquito midgut epithelium to reach its basal side, where they develop into oocysts. *Plasmodium* encounters several obstacles at each of its developmental stages and spatial transitions within the mosquito. One of the major barriers is the midgut epithelium, within which *Plasmodium* is attacked by the mosquito's immune system. These defense reactions involve a variety of immune components that reduce the parasite population by several-fold and have mainly been described at the stage of ookinete invasion and beyond [1].

Survival of ookinetes in the midgut epithelium has been shown to depend on the action of agonists and antagonists. Recent studies have identified two infection-inducible putative pattern recognition receptors, Tep1 and LRIM1, that can mediate killing of ookinetes in the midgut epithelium; in contrast, two c-type lectins, CTL4 and CTLMA2, can protect the ookinetes from destruction [2]. Other known factors with activity against the midgut stages of *Plasmodium* include nitric oxide, the antimicrobial peptides gambicin and cecropin, and an apolipophorin precursor RFABG [3–7]. Recent studies have linked the A. gambiae NF-kappaB–like transcription factor REL2 and adaptor protein IMD to the defense against P. berghei and thereby established a role for the putative IMD pathway in anti-*Plasmodium* defense [8]. *Plasmodium* infection will also affect a variety of other biological processes in addition to those linked to the immune response [7].

Activation of immune gene transcription has also been documented prior to ookinete invasion, suggesting that other constituents of malaria-infected blood are sensed by the immune surveillance system and can thereby elicit immune responses [9]. The ingested malaria-infected blood differs from noninfected blood in a number of ways, including the presence of blood-stage and gametocyte-stage *Plasmodium* and their metabolites, and of vertebrate infection-responsive molecules, as well as higher free-radical concentrations [10,11]. These biochemical and cellular factors can be expected to influence a variety of biological processes in the mosquito, including the activation of immune responses [3]. Midgut invasion by ookinetes is accompanied by apoptosis of the invaded epithelial cells, which are expelled into the lumen [12].

Most studies addressing A. gambiae responses to *Plasmodium* infection have utilized the rodent Plasmodium berghei model system, which is more amenable to experimental manipulation than is the human parasite P. falciparum. However, A. gambiae is not the natural vector of *P. berghei,* for which it is significantly more permissive than for *P. falciparum: P. berghei* frequently produces more than 300 oocysts on the midgut epithelium, while P. falciparum rarely produces more than two to five oocysts, either under laboratory conditions or in nature [13,14]. At present, the dependence of these infection levels on the mosquito's immune responses or other factors is not known. At the cellular level, the ookinete invasion route and midgut epithelial response appear to be similar for the two parasite species, but the process may proceed more rapidly for P. falciparum at some stages because of its ~3 °C higher temperature of infection [15,16]. The mosquito's immune system is most likely predominantly devoted to combating the bacterial and fungal pathogens present in its external environment and intestinal flora, and it is unclear whether defense mechanisms have evolved that are specific for *Plasmodium* [17,18].

We have used an experimental design involving wild-type (wt) and transgenic mutant parasite strains of both P. falciparum and P. berghei species to analyze and compare A. gambiae responses, at the global transcript level, to the invading ookinetes of both human and rodent parasite species and to human malaria-infected blood lacking invasive ookinetes. A similar strategy has been used previously [7] to analyze midgut transcriptomic responses to P. berghei ookinete invasion; however, this previous study was more broadly focused on midgut responses relating to a variety of biological processes rather than the specific immune responses to the parasite. We have furthermore compared the anti-*Plasmodium* immune responses with those acting against bacteria with RNAi gene-silencing assays. Our analysis provides insight into the species specificity of the immune defense against *Plasmodium* and its relationship to the mosquito's antibacterial defense system.

## Results

### Responses to Invasion of the Midgut Epithelium by *P. falciparum* and P. berghei Ookinetes

**Figure 1:**
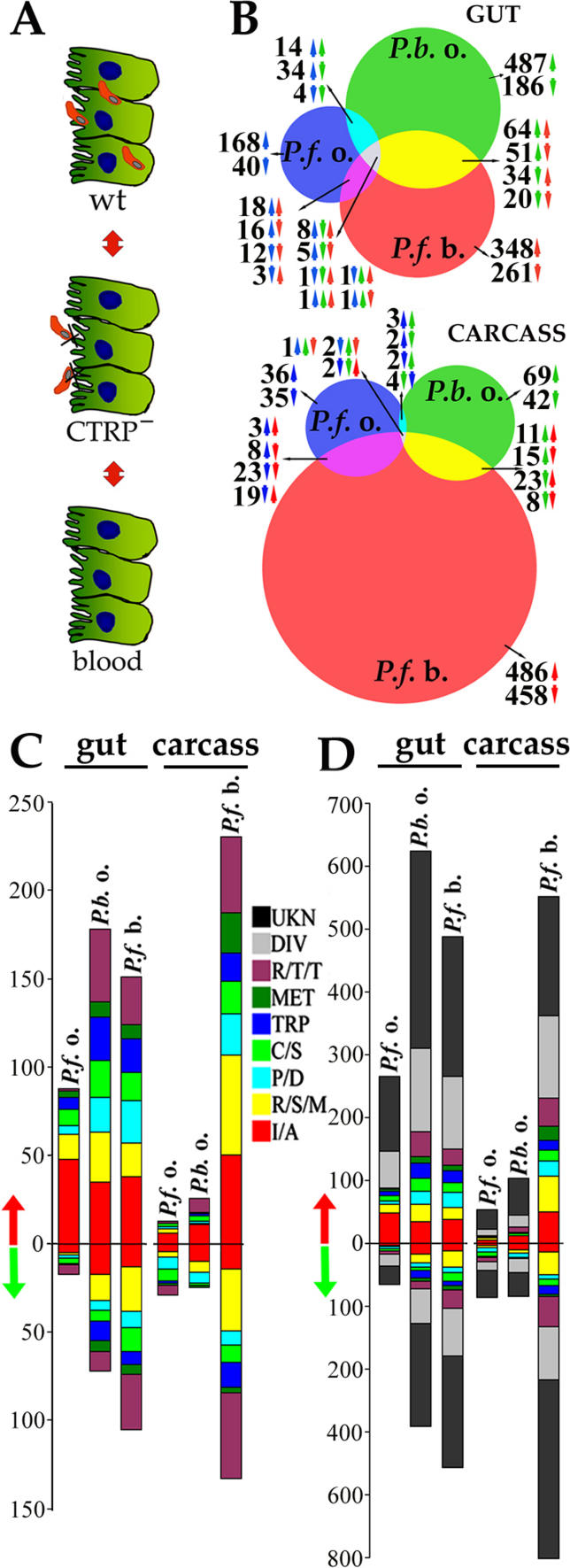
Experimental Design and Global Gene Expression Patterns at the Different Conditions of Infection (A) Model outlining the experimental design for assessing responses to the invading ookinetes (indicated as *P.f.* ookinete [*P.f.* o.] and *P.b*. ookinete [*P.b.* o.] in (B) and (C) by comparing transcription between mosquitoes fed on blood infected with a wt *Plasmodium* strain and those fed on blood infected with the invasion-incapable mutant *Plasmodium* strain CTRP^−^. Responses to infected blood (indicated as *P.f*. blood in [B] and [C]) in the absence of ookinete invasion were assessed by comparing gene expression between mosquitoes fed on blood infected with the P. falciparum invasion-incapable mutant and mosquitoes fed on noninfected (no *Plasmodium*) blood. (B) Gene regulation in midgut and carcass tissues triggered by P. falciparum ookinete invasion (*P.f.* ookinete), P. berghei ookinete invasion (*P.b.* ookinete), and P. falciparum strain CTRP^−^-infected blood lacking invasive ookinetes (*P.f.* blood). Colored arrows indicate genes that are up- or down-regulated in the various unique and overlapping sections. (C) Proportions and numbers of genes belonging to distinct functional classes which were up- or down-regulated by the various stimuli in the gut and carcass tissues. DIV: diverse; R/T/T: replication, transcription, translation; MET: metabolism; TRP: transport; CY/ST: cytoskeletal, structural; PR/DI: proteolysis, digestion; MIT: mitochondrial; RE/ST: oxidoreductive, stress-related; APO: apoptosis; P/A: putative immunity and apoptosis. Gene functions were predicted based on Gene Ontology data and manual sequence homology searches. (D) Same as in (C), but also including genes of diverse functions (DIV) and unknown functions (UKN).

Ookinete-stage *Plasmodium* invades the mosquito midgut epithelium over a 10-h period beginning about 20 h after ingestion of infected blood. The peak of P. berghei (21 °C) and P. falciparum (24 °C) ookinete invasion occurs at 24–26 h after ingestion [17,19]. To assess the impact of ookinete midgut invasion on the mosquito transcriptome, we compared gene expression in the gut and carcass tissues of mosquitoes that had fed on a wt *Plasmodium*-infected blood and those that had fed on blood infected with an invasion-incapable circumsporozoite- and TRAP-related protein (CTRP) knockout (CTRP^−^) Plasmodium mutants. The experimental strategy used to assay the mosquito responses to ookinete invasion and infected blood is presented in Figure 1A. Identical assays with wt *Plasmodium* and CTRP knockout mutants were conducted with P. falciparum and *P. berghei,* allowing comparison of the mosquito responses to midgut invasion by ookinetes of the two parasite species. The entire predicted A. gambiae transcriptome was screened in these assays using a 60-mer oligonucleotide microarray (Agilent Technologies, Palo Alto, California, United States). The gene regulation threshold cutoff was determined to be 1.74-fold, which corresponds to 0.8 in log2 scale, according to [20].

P. falciparum ookinete invasion triggered the regulation of 471 genes in the midgut and carcass tissues, corresponding to ~3.4% of the mosquito transcriptome; P. berghei ookinete invasion elicited changes in the expression of 1,102 genes, corresponding to 8.1% of the mosquito transcriptome. The mosquito midgut and carcass responses to P. falciparum and P. berghei ookinete invasion were remarkably divergent, with only limited overlap in gene expression (Figure 1B–1D); for example, the overlap in transcriptional responses to the two parasite species in the midgut involved only 16 induced and five repressed genes. Midgut invasion by the P. falciparum ookinetes elicited less profound gene regulation than *P.berghei.* Specifically, P. falciparum induced 265 genes and repressed 65 genes in the midgut, while P. berghei invasion produced alterations in more than three times as many genes, inducing 623 and repressing 292 (Figure 1B–D).

P. falciparum induced more putative immune genes in the mosquito midgut than did P. berghei (48 versus 35; Figure 1C and 1D). It is possible that a proportion of the P. berghei induced genes is implicated in as-yet-unknown defense mechanisms and has therefore not been assigned to the immunity class. In general, fewer genes were induced or repressed in the carcass by ookinete invasion of the midgut (141 for P. falciparum versus 187 for *P. berghei;* Figure 1B–D). Quantitative RT-PCR assays of the expression patterns of 15 control genes, under several experimental conditions, validated microarray assays (Figure S1; Table S4; Protocol S1).

### Responses to Malaria-Infected Blood in the Absence of Midgut Invasion

The impact on the mosquito midgut and carcass transcriptomes of P. falciparum–infected blood that lacked invasive ookinetes was investigated by comparing gene expression between mosquitoes that had fed on blood infected with the CTRP knockout mutant P. falciparum strain (incapable of ookinete invasion; used as experimental sample) and mosquitoes fed on non-*Plasmodium*–infected blood (used as a reference sample) at 24 h after ingestion, when ookinete invasion of the midgut normally takes place (Figure 1A). The mosquitoes were clearly capable of sensing and discriminating between infected blood and non-infected blood.

Ingestion of CTRP knockout–infected blood elicited changes in the expression of as many as 1,896 genes in the midgut and carcass tissues, corresponding to approximately 14% of the A. gambiae transcriptome (Figure 1B–1D; Tables S1 and S2). The magnitude of the gene regulation in the midgut in response to P. falciparum CTRP knockout mutant-infected blood was comparable to that induced by P. berghei ookinete invasion (844 versus 915 genes). Six times as many genes (1,052) were affected in the carcass tissues as were regulated by P. berghei ookinete invasion (187). The 487 induced and 357 repressed genes in the midgut tissue represented a variety of functional classes, including 51 putative immune genes.

### Molecular Immune Responses

Invasion of the midgut epithelium by P. falciparum and P. berghei ookinetes as well as infected blood containing noninvasive ookinetes induced putative immune-related genes. Genes that showed significant up- or down-regulation (at least 1.74-fold) in at least one experiment were grouped into clusters according to the specificity of their regulation for the midgut tissue and/or carcass. We divided these 157 transcripts into seven groups based on their expression patterns and tissue specificity (Table 1).

**Table 1:**
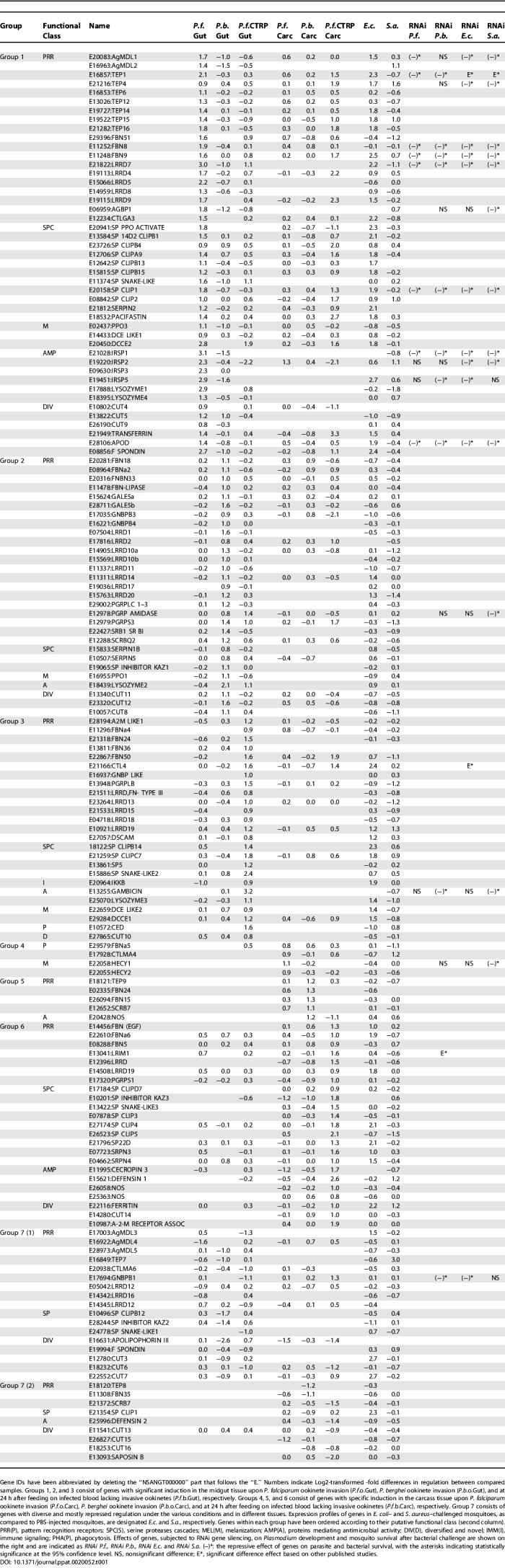
Grouping of Putative Immune Genes into Seven Distinct Groups, According to their Regulatory Significance in the Midgut and Carcass Tissue

Groups 1, 2, and 3 consisted of 45 genes that were induced in the midgut by P. falciparum ookinete invasion, 29 genes induced by P. berghei ookinete invasion, and 25 genes induced by ingested infected blood lacking invasive ookinetes (the CTRP mutant), respectively. While P. falciparum ookinete invasion induced almost twice as many immune genes as did the other two infection conditions, the distribution of functional classes was quite similar among the three groups. Almost half of the genes in each group encoded putative pattern recognition receptors that belong to the MD2-like protein family (AgMDL), the fibrinogen domain immunolectin family (FBN), the thioester-containing protein family (Tep), the Gram-negative bacteria binding protein family (GNBP), the peptidoglycan recognition protein family (PGRP), the C-type lectin family (CTL), gal-lectin family (GALE), the scavenger receptor family, the leucine-rich repeat domain protein family (LRRD), and the bacteria recognition family (AGBP) [18,21–29]. We suggest that some of these proteins are required for the recognition of *Plasmodium* and subsequent activation of defense reactions. Other transcripts in groups 1–3 encoded immunity-related serine proteases and serine protease inhibitors that are most likely involved in immune signal amplification cascades. Several transcripts encoding enzymes involved in melanization reactions were induced in the midgut. Finally, lysozymes and the mosquito-specific antimicrobial peptide gambicin (E13255) were induced.

Groups 4, 5, and 6 consisted of immune genes that were induced in the carcass tissues, and group 7 consisted of a variety of immune genes that were mostly repressed under the various experimental conditions.

The proportion of genes induced in invaded cells may be larger than that seen for the entire midgut tissue, because a relatively small number of cells is invaded by ookinetes; we therefore suspect that we might have missed genes that are highly expressed in invaded cells but are diluted out by the rest of the midgut. Hemocytes that are attached to the midgut wall but difficult to separate by dissection have been shown to express Tepl, LRIM1, and other effectors that act against *Plasmodium* in the midgut [2,30]. Some of the gene activation that we detected in infected midgut samples is therefore likely to be derived from hemocytes. Preliminary studies have shown that as many as 30 of the 157 putative immune genes listed in Table 1 are highly expressed in hemocytes (Strand and Dimopoulos, unpublished data). Many of the immune genes identified here have also been found to be induced in the midguts of A. gambiae refractory L3–5 and susceptible G3 and 4A-RR strain mosquitoes upon P. berghei infection [7,17,31].

Our analyses identified several novel infection-responsive genes that we suggest are components of the mosquito's immune system; these are discussed in detail in the Supporting Information (Protocol S1). Data concerning other biological processes in the mosquito that are affected by ookinete invasion of the midgut and ingestion of malaria-infected blood are detailed in the Supporting Information (Protocol S1).

### Determinants of Mosquito Resistance to Infection

We assume that the mosquito's immune response is largely regulated at the level of mRNA abundance [2,7,17,32]. Based on this assumption, we predict that many of the infection-stimulated immunity-related genes are necessary to defend against *Plasmodium.* We took an RNAi-based reverse genetic approach to test the role these induced genes played in fighting a *Plasmodium* infection. Transcripts of selected genes were targeted with double-strand RNAs (dsRNAs) prior to experimental infection with the pathogens to assess the potential effects of the transcript depletion on the infection phenotype. Nineteen genes were selected on the basis of their expression patterns and putative functions in innate immunity, as predicted by their sequences. The efficiency of RNAi-mediated transcript depletion was verified by quantitative real-time RT-PCR (Table S6 in Results S1). These 19 genes encode putative pattern recognition receptors (AgMDL1, TEP1, TEP4, FBN8, FBN9, FBN39 [E21380]), LRRD7, AGPB1, PGRP-AMIDASE, GNBPB1; the serine protease SPCLIP1; the antimicrobial peptide gambicin; the hemocyanin HECY1, the apolipoprotein APOD; the kininogen KIN1; and four short secreted peptides, IRSP1, IRPS2, IRSP3, and IRSP5. Two genes were tested but did not display any significant effects (*KIN1* [E14131] and *IRSP3* [E09630]) (unpublished data). *Tep1* was used as a positive control as silencing of *Tep1* in a susceptible G3 A. gambiae strain results in an up to 7-fold increase in P. berghei infection [30]. All genes were used in challenge experiments with P. berghei while only a subset of genes were tested with P. falciparum because of the difficulty of performing these experiments. Detailed information on the genes selected for gene silencing assays is presented in the Supporting Information (Protocol S1).

### Anti-*Plasmodium* Defense Activities

**Figure 2:**
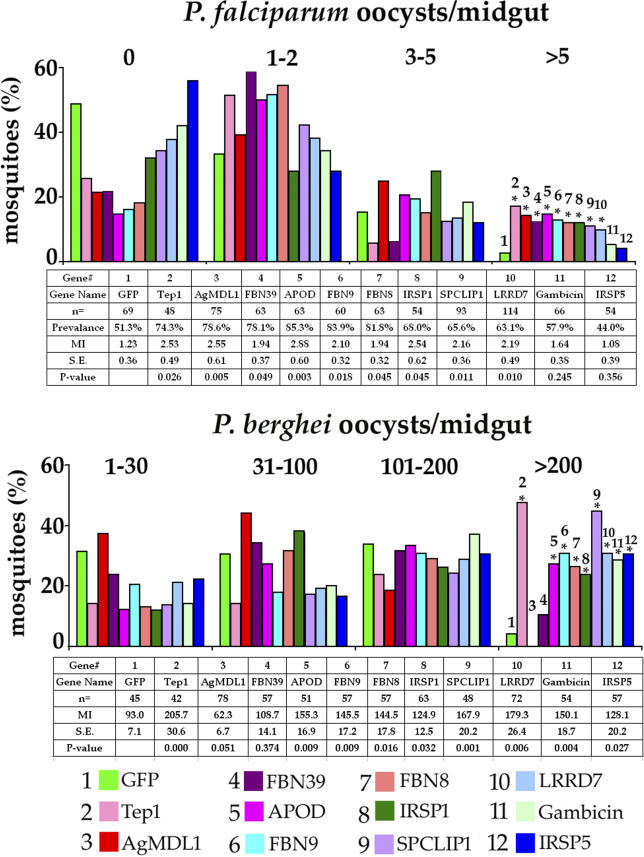
Effects of Gene Silencing of 11 Selected Putative Immune Genes on P. falciparum and P. berghei Infection The gene silencing efficiency values (KD %) are displayed in Table S6. The frequency distribution of oocysts pooled from three independent assays is displayed, with bars indicating the percentile of mosquitoes with the corresponding oocyst number in the range indicated on the *x*-axis. Equal numbers of midguts from all three experiments in each dataset were pooled. Bars with asterisks indicate the statistically significant differences at the 95% confidence level, based on the *p* value from two independent probability tests, the KS and Mann-Whitney tests (Tables S5 and S6). n: total midguts assayed; MI: mean intensity of infection (oocysts number); S.E.: standard error of mean intensity; *p* value: from Mann-Whitney test.

**Figure 3:**
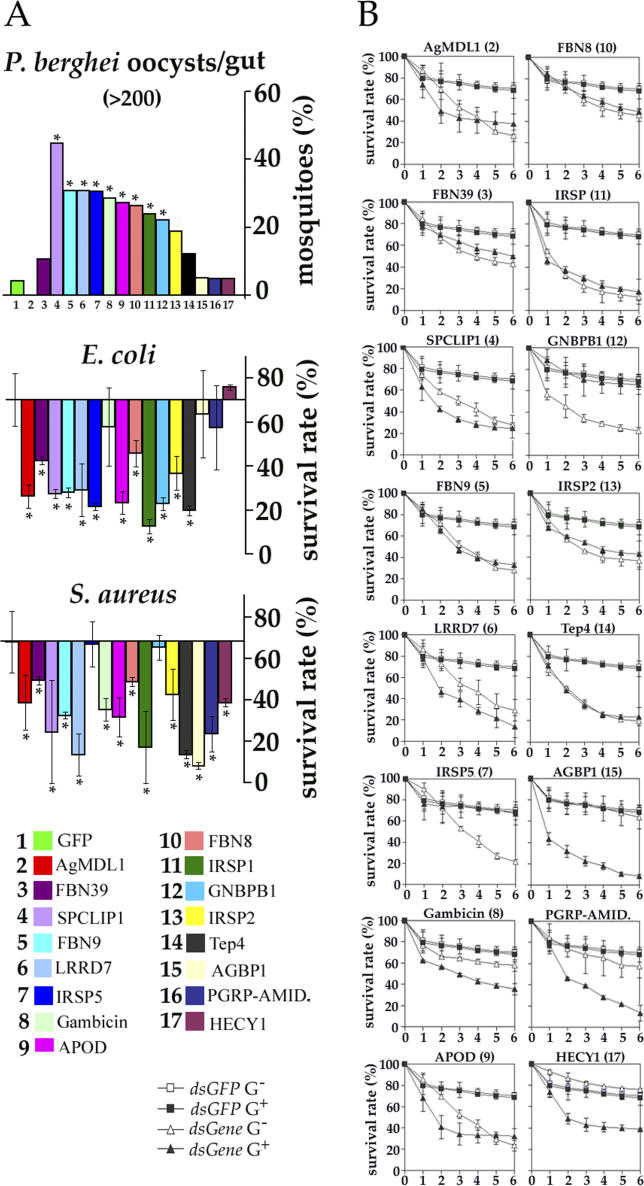
Comparison of Anti-*Plasmodium* and Antibacterial Activities for 16 Selected Immune Genes (A) Effect of gene silencing on P. berghei development, as described in Table S6. For ease of comparison, only the mosquito portions with the highest P. berghei oocyst numbers (>200) are presented. The effect of gene silencing on bacterial infection is presented as the mosquito survival at d 6 after challenge with E. coli and *S. aureus.* After 6 d, the survival rates stabilized and did not change significantly until age-related mortality ensued. The baseline survival rate was set to that of the challenged GFP dsRNA-treated control mosquitoes (~70%). Standard error bars with asterisks indicate the results of two-way analysis of variance, with *p* < 0.05 considered statistically significant. The gene names are numbered for ease of comparison. (B) Mosquito survival rates for each silenced gene after challenge with E. coli and *S. aureus.* The numbers in parenthesis correspond to the numbers in (A). Open squares, *dsGFP* control-treated mosquitoes challenged with E. coli; solid squares, *dsGFP* control–treated mosquitoes challenged with *E. coli;* open triangles, gene-silenced mosquitoes challenged with *E. coli;* and solid triangles, gene-silenced mosquitoes challenged with *S. aureus.* Standard error bars from three replicate experiments are included for each time point.

Silencing of 11 of the transcripts *(Tep1, AgMDL1, FBN8, FBN9, FBN39, SPCLIP1, APOD, IRSP1, IRSP5, LRRD7, gambicin)* resulted in increased *Plasmodium* levels, in both the present work and previous studies [22,23,30,33–36] (Dimopoulos, unpublished data; Figures 2 and 3; Tables S5 and S6). The effects of gene silencing on the susceptibility to *Plasmodium* infection were evident in the differences between the gene-silenced and green fluorescent protein (GFP) dsRNA-treated control mosquitoes in terms of the proportion of mosquitoes exhibiting a very low number of oocysts and the proportion exhibiting exceptionally high oocyst numbers (Figures 2 and 3). Although most of these anti-*Plasmodium* factors were induced in the midgut tissue, several were also induced in carcass tissues (Table 1).

Seven genes that strongly influenced both P. falciparum and P. berghei development, *Tep1, APOD, FBN8, FBN9, SPCLIP1, IRSP1,* and *LRRD7,* were all induced in the mosquito midgut upon P. falciparum ookinete invasion but not in P. berghei–infected mosquitoes (Figure 2 and Table 1). Silencing of *AgMDL1* and *FBN39* showed a more pronounced effect on P. falciparum development, while silencing of the gene encoding the antimicrobial peptide *gambicin* and an infection-responsive secreted peptide gene *IRSP5* had a specific effect on the resistance to P. berghei infection.

Though P. falciparum appeared to elicit a stronger immune response at the gene transcript level than *P. berghei,* knockdown of most of the genes that were specifically induced by P. falciparum ookinete invasion surprisingly also affected P. berghei levels.

### Relationships between Anti-*Plasmodium* and Antibacterial Immune Responses

To assess the relationships between the transcriptional immune responses to *Plasmodium* and other immune challenges, we compared the gene expression in mosquitoes injected with Escherichia coli and Staphylococcus aureus to *Plasmodium*-infected mosquitoes. Of the 132 genes that were induced by *Plasmodium* ookinete invasion or infected blood, 49 were also induced by E. coli challenge, and 12 genes were induced by S. aureus challenge. The E. coli infection-responsive transcripts included eight genes *(Tep1, FBN9, FBN39, LRRD7, CTL4, SPCLIP1, IRSP5,* and *APOD)* that can influence *Plasmodium* development and resistance to bacterial challenge (Table 1 and Figures 2 and 3; presented below). The overlap between *Plasmodium-* and bacteria-elicited immune gene regulation supports the hypothesis that the mosquito is utilizing some of the same immune pathways and mechanisms for defense against these two classes of pathogen.

All 19 genes that were tested for their effect on P. berghei development through RNAi-mediated depletion were also able to influence mosquito resistance to bacterial challenge and can therefore be considered as components of the antibacterial defense. The anti-*Plasmodium* factors Tep1, gambicin, and NOS have been shown in previous studies to mediate anti-*Plasmodium* and antibacterial defenses [3,4,30]. Five genes encoding IRSP2, Tep4, PGRP-AMIDASE, AGBP1, and HECY1 were specific for antibacterial defense and had no significant effect on the *Plasmodium* infection (Table 1 and Figure 3). Three genes that had effects on mosquito survival upon S. aureus challenge, *PGRP-AMDISAE, AGBP1,* and *HECY1,* had no significant effects on survival after E. coli challenge or infection by *Plasmodium,* whereas two genes, *IRSP5* and *GNBPB1,* were more specific for E. coli and P. berghei. Only one gene, *gambicin,* could influence resistance to both P. berghei and *S. aureus.*

## Discussion

Both *Plasmodium* ookinete invasion and other factors in infected blood serve as triggers of the immune and other responses by the *Anopheles* mosquito. The diverse midgut responses to P. falciparum and P. berghei infection can be attributed to differences in infection level and in the biology of interaction between the two parasite species and the mosquito [13,37]. Achieving comparable infection levels for the human and rodent parasites in *A. gambiae,* through artificial manipulation of infections, would be difficult and not appropriate for the scope of this study, which addresses the relevance of analyses with a laboratory experimental model. The unnaturally high infection levels in P. berghei are useful for the experimental analysis of gene expression patterns that may be undetectable at the low infection levels of *P. falciparum.* An example of this phenomenon is the induction of cytoskeletal genes in this and other studies upon P. berghei infection, that have been shown to act as both agonists and antagonists of *Plasmodium* [7,38]. The smaller number of induced putative immune genes upon P. berghei infection may indicate that the mosquito's immune surveillance system is more capable of sensing P. falciparum, or P. berghei may in some way suppress the mosquito's immune response, and that could partly explain the significantly higher infection levels of the rodent parasite in *A. gambiae.*

While the midgut is the primary site of response to the invading ookinetes, the observed changes in gene expression in the carcass tissues, at a time point when the ookinetes are in the midgut epithelium, most likely reflect intertissue signaling from the midgut epithelium to hemocytes and fat body cells, possibly through cytokine-like molecules. Alternatively, parasite-derived molecules that diffuse into the hemolymph may affect mosquito biological processes in the carcass tissues of the mosquito [24].

The broader effect of infected blood on gene regulation, as compared to ookinete invasion of the midgut, can be attributed to the exposure of all the midgut cells to the infected blood components, while only a subset of cells are invaded by the ookinetes, and indicates the extensive qualitative differences between infected and noninfected blood [10,11]. The capacity to mount an immune response to infected blood, in the absence of ookinete invasion, is likely beneficial in controlling *Plasmodium* infection. This strategy would allow for enrichment of anti-*Plasmodium* factors prior to epithelial invasion. P. falciparum glycosylphosphatidylinositols in malaria-infected blood have been shown to act as potent elicitors of immune responses [3,11]. It is also possible that the immune response acts against parasite stages in the midgut lumen prior to invasion. For example, the antimicrobial peptide gene *Gambicin,* which is induced by infected blood in the absence of invasion, has been shown to be highly expressed in the cardia tissue of the anterior midgut, which is not invaded by *Plasmodium* [4] (Dimopoulos lab, unpublished data). From the cardia, gambicin and other effectors may be blended into the blood meal, where they can limit bacterial growth and attack *Plasmodium* in the midgut lumen.

A recent study by Vlachou and coworkers utilized an expressed sequence tag (EST)–based cDNA microarray comprising approximately 8,000 A. gambiae genes to assay midgut gene expression responses to P. berghei ookinete invasion [7]. Surprisingly, of the 914 P. berghei regulated genes identified in the present study and the 346 regulated genes with accession numbers from the previous study, only 25 genes showed similar regulation in the two studies. These differences can presumably be attributed to the differences in the two experimental systems: we utilized different A. gambiae and P. berghei strains, and a different type of microarray [2]. Even small differences in rearing and infection conditions could also have affected the responses [7,31]. A previous study has also documented significant differences in transcriptional infection responses between different A. gambiae lab strains upon challenge with the same pathogens [31]**.** A. gambiae most likely possesses a variety of anti-*Plasmodium* defense mechanisms, and different strains may differ in their usage of these defenses. For instance, one genetically selected A. gambiae strain melanotically encapsulates the invading ookinetes, while another selected strain lyses the ookinetes in the midgut epithelium [39,40].

The high proportion of tested genes that had an effect on *Plasmodium* and bacterial infection in this study can be explained by the targeted selection of putative immune genes with a bias towards P. falciparum infection-induced transcripts. RNAi gene-silencing assays in A. gambiae are based on the direct injection of gene-specific dsRNAs into the hemolymph, which is in direct contact with the fatbody, midgut, hemocytes, and other tissues [41]. The genes we examined are expressed in different tissues and even in different cell types within the same tissues [42]. Consequently, the gene knockdown phenotypes may to some extent also reflect the efficacy of dsRNA uptake and gene silencing of different tissues and cell types in addition to specific gene functions. Several anti-*Plasmodium* genes were expressed in carcass tissues and hemocytes (Strand and Dimopoulos, unpublished data) (Table 1) [2,30]. These factors are likely to be present in the hemolymph, from which they are able to attack the midgut-stage *Plasmodia* on the basal side of the midgut or even within the epithelium by diffusion through the basal labyrinth, which is a channel system extending into the cells [43]. The extensive overlap between gene effects on P. falciparum and on P. berghei development suggest that the mosquito's defense mechanisms are quite universal for different *Plasmodium* species, although species-specific defense mechanisms also exist.

**Figure 4:**
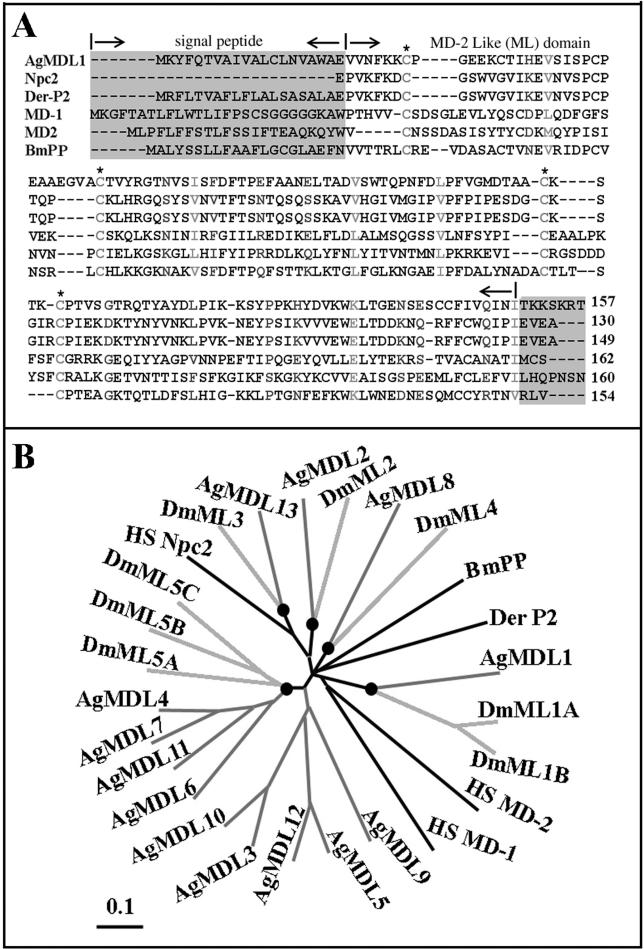
AgMDL Gene Family (A) A. gambiae MD2-like genes encode proteins ranging from 130 to 162 amino acids and include signal peptides and an ML lipid recognition domain. Alignment of AgMDL1 with the human homologues MD1, MD2, and Npc2, the mite allergen Der-P2, and the Bombyx mori promotor protein (BmPP). Two conserved cysteines, Cys95 and Cys105, that are essential for binding to TLR4 are indicated with asterisks. (B) Phylogenetic tree of MD2-like proteins from *A. gambiae, D. melanogaster, B. mori,* and humans. 1:1 orthologs and ortholog groups are highlighted with filled circles. Ag, *Anopheles gambiae;* Dm, D. melanogaster. The accession numbers for these genes are listed in Table S7.

Silencing of putative anti-*Plasmodium* factors resulted in an increase in P. falciparum levels of up to 6-fold; in terms of mean oocyst numbers, this increased level is significantly lower than the infection level of P. berghei in non–gene-silenced mosquitoes (Figure 2; Tables S5 and S6). The lower infection level of the human parasite species is therefore attributable to nonimmunity related factors or, alternatively but less likely, to immune factors that were not identified or tested in the present study. It is not yet clear how many of the different anti-*Plasmodium* factors function together in the same mechanism or pathway, and which may be acting independently. Some of the tested genes may participate in anti-*Plasmodium* defense but not be essential because their function is redundant with that of other immune genes. The infection phenotype of such genes after RNAi knockdown will therefore not differ from that in untreated mosquitoes. Genes with differential effects on infection with different pathogens, such as *gambicin* and *AgMDL1,* could reasonably be expected to act in different defense mechanisms. Future analyses will address the relations and hierarchies of these anti-*Plasmodium* factors in the fight against malaria*.* Of particular interest is the anti–P. falciparum–specific activity of the novel mosquito immune factor *AgMDL1*, which may act as an immune pathway activator similarly to its vertebrate homologue (Figure 4 and Protocol S1) [21,28].

The genes displaying anti-*Plasmodium* activity also influenced the mosquito's resistance to bacterial infection, while several genes with an effect on resistance to bacterial infection did not influence *Plasmodium* development. These findings suggest the mosquito is mainly employing its antimicrobial defense system in the fight against malaria. Although certain immune gene allele frequencies have been correlated with *Plasmodium* exposure in the field, there is little reason to believe that the mosquito would have undergone major adaptations to malaria and evolved highly specific anti-*Plasmodium* defense mechanisms [44,45]. In nature, bacteria and fungi are most likely the major pathogens to which the mosquitoes are continuously exposed. In contrast, exposure to *Plasmodium* is seasonal and usually very low-level, rarely exceeding 25% of the infected mosquitoes in a given population [46]. A recent study has linked the A. gambiae anti-*Plasmodium* defense to the REL2F factor, which also mediates activation of the defense against S. aureus [8]. Up-regulation of the antibacterial-specific genes *IRSP2, Tep4, PGRP-AMIDASE, AGBP1,* and *HECY1* after *Plasmodium* infection may be attributed to concomitant microbial infections of the midgut epithelium; it is very likely that the ookinete invasion of the epithelium facilitates exposure to bacteria and bacterial components such as lipopolysaccharide and peptidoglycan (Table 1). Through this mode of gene induction, the bacteria may participate in boosting the mosquito's anti-*Plasmodium* defense. Previous studies have shown that antibiotic-treated mosquitoes, with significantly reduced microbial midgut flora, express lower levels of immune genes and are more susceptible to *Plasmodium* infection [19,47]. Conversely, mosquitoes that have been challenged with bacteria are more resistant to *Plasmodium* infection [48].

This study suggests that P. berghei is a useful malaria model for studying anti-*Plasmodium* gene function but may be less relevant as a model for studying transcriptional immune responses to ookinete invasion of the midgut epithelium. A comprehensive understanding of the interactions between *Anopheles* and *Plasmodium* can lead to the development of new strategies for controlling malaria, based on the mosquito's own defense against the parasite [49].

## Materials and Methods

### Mosquito rearing and infection assays.

A. gambiae Keele strain mosquitoes were maintained on sugar solution at 27 °C and 70% humidity with a 12-h light/dark cycle according to standard rearing procedures [50]. For microarray assays, the carcasses and midguts from approximately 40 mosquitoes were dissected on ice 24 h after ingestion of blood infected with the wt Anka 2.34 or CTRP^−^ P. berghei strain, or the wt NF54 or CTRP^−^ P. falciparum strain, or noninfected human blood. P. falciparum gametocyte cultures were prepared as previously described, and mosquitoes were fed on cultures through a membrane feeder at 27 °C and then maintained at 24 °C [51]. *P. berghei* infections were done at 21 °C as previously described [7]. Mosquito midguts were dissected at 7–8 d after feeding and stained with 0.2% mercurochrome. Oocyst numbers per midgut were determined using a light-contrast microscope (Olympus, Tokyo, Japan). P. berghei infections with gene-silenced (RNAi) mosquitoes were performed with a transgenic *GFP P. berghei* strain and infection phenotypes were determined as previously described [7]. For preparation of bacterially challenged samples for microarray analyses, 4-d-old female mosquitoes were first injected with approximately 20,000 heat-inactivated E. coli or S. aureus and approximately 20 whole mosquitoes were collected 4 h after challenge [52]. For bacterial challenge of gene-silenced (RNAi) mosquitoes, E. coli and S. aureus were cultured in LB broth overnight, then washed three times with phosphate-buffered saline (PBS) before being resuspended in PBS. Approximately 27,000 live E. coli or 55,000 S. aureus in a 50-nl PBS suspension were injected into the mosquito hemolymph 4 d after the dsRNA injections. RNA was extracted from dissected tissues or whole mosquitoes by using the RNeasy kit (Qiagen, Valencia, California, United States). Quantification of RNA was performed using a Biophotometer (Eppendorf, Hamburg, Germany) spectrophotometer, and quality assessment was determined by RNA Nano LabChip analysis on an Agilent Bioanalyzer 2100.

### Probe sequence design and microarray construction.

The release 2a A. gambiae sequences were retrieved from Ensembl (http://www.ensembl.org/Anopheles_gambiae). These sequences were predicted using a combination of ab initio, EST, and protein similarity–based methods [53–55]. The transcripts were annotated with the EnsMart utility (http://www.ensmbl.org) [56,57]. Oligonucleotides (60 mer) for the 14,180 predicted A. gambiae transcripts that corresponded to 13,118 genes were designed using the Oligo Picky software according to the software developer's instructions [58]. Oligonucleotide sequences were designed to be complementary to regions within 1 kb of the 3′ untranslated region of transcripts and had a minimal sequence identity overlap with nontarget transcript sequences. Microarrays were constructed through in situ synthesis of oligonucleotides on glass slides by Agilent Technologies.

### Microarray analysis.

Fluorochrome-labeled cRNA probes were synthesized from 2–3 μg of RNA using the Agilent Technologies low-input linear amplification RNA labeling kit according to the manufacturer's instructions. Probe quantity was determined with a Biophotometer spectrophotometer, and 16-h hybridizations were performed with the Agilent Technologies in situ hybridization kit according to the manufacturer's instructions. After washes, the prescribed microarrays were instantaneously dried with pressurized air. Microarrays were scanned with an Axon GenePix 4200AL scanner using a 10 μm pixel size (Axon Instruments, Union City, California, United States). Laser power was set to 100%, and the photomultiplier tube voltage was adjusted to maximize effective dynamic range and minimize pixel saturation. The spot size, location, and quality were determined using GenePix software Pro 6.0 algorithms, and potential misidentifications of spot locations and quality were corrected manually. Scan images were analyzed, and Cy5 and Cy3 signal and ratio values were obtained using Genepix software. The minimum signal intensity was set to 200 fluorescent units, and the signal-to-background ratio cutoff was set to 2.0 for both Cy5 and Cy3 channels. Three or four biological replicates were performed for each experimental set. The background-subtracted median fluorescent values for good spots (no bad, missing, absent, or not-found flags) were normalized according to a LOWESS normalization method, and Cy5/Cy3 ratios from replicate assays were subjected to *t* tests at a significance level of *p* ≤ 0.05 using TIGR MIDAS and MeV software [59]. For genes with significant *p* values in one experimental set, the expression values from other experimental sets were included when the direction of regulation in all the replicate assays was the same and within a regulation range of ≤ 0.5-fold. Expression data from all replicate assays were averaged with the GEPAS microarray preprocessing software prior to logarithm (base 2) transformation [60]. Self–self hybridizations were used to determine a cutoff value for the significance of gene regulation of 0.8 in log2 scale, which corresponds to 1.74-fold regulation according to previously established methodology [20]. The false discovery rate was therefore 0.027% (three standard deviations). Microarray-assayed gene expression of 15 genes was further validated with quantitative RT-PCR and showed a high degree of correlation (Pearson correlation coefficient *p* = 0.86; best-fit linear-regression *R^2^* = 0.75; and the slope of the regression line *m* = 0.996) for 15 tested genes (Figure S1).

### Real-time quantitative PCR.

RNA samples were treated with Turbo DNase (Ambion, Austin, Texas, United States) and reverse-transcribed using Superscript III (Invitrogen, Carlsbad, California, United States) with random hexamers. Real-time quantification was performed using the QuantiTect SYBR Green PCR Kit (Qiagen) and ABI Detection System ABI Prism 7000 (Applied Biosystems, Foster City, California, United States). All PCR reactions were performed in triplicate. Specificity of the PCR reactions was assessed by analysis of melting curves for each data point. The ribosomal protein S7 gene was used for normalization of cDNA templates. Primer sequences are listed in Table S3.

### RNAi gene-silencing assays.

Sense and antisense RNAs were synthesized from PCR-amplified gene fragments using the T7 Megascript kit (Ambion). The sequences of the primers are listed in Table S3. About 69 nl of dsRNA*s* (3 μg/μl) in water was introduced into the thorax of cold-anesthetized 4-d-old female mosquitoes using a nano-injector (Nanoject; Drummond Scientific, Broomall, Pennsylvania, United States) with a glass capillary needle according to established methodology [41]. For gene-silencing assays, 80 4-d-old female mosquitoes were injected, in parallel, with *GFP* dsRNA as a control group or with target gene–specific dsRNA for the experimental group. Gene silencing was verified 3 to 4 d after dsRNA injection by real-time quantitative RT-PCR, done in triplicate, with the A. gambiae ribosomal *S7* gene as the internal control for normalization (Table S6). The primers for silencing verification are listed in Table S3. For *Plasmodium* infection assays, 3–4 d after dsRNA injection, at least 50 control (GFP dsRNA–injected) and 50 experimental (gene dsRNA–injected) mosquitoes were fed on the same P. berghei–GFP strain–infected mouse or the same NF54 P. falciparum culture; 24 h later, the unfed mosquitoes were removed [7]. Mosquito midguts were dissected at 7–8 d after feeding and stained with 0.2% mercurochrome. Oocyst numbers per midgut were determined using a light-contrast microscope (Olympus). Infection phenotypes of the transgenic *GFP P. berghei*–infected mosquitoes were determined as previously described [7]. The mean number of oocysts per midgut was calculated for each tested gene and for *GFP* dsRNA–injected control mosquitoes. The results for equal numbers of midguts from all three independent biological replicates were pooled. Because of the lower P. falciparum infection levels, the Kolmogorov-Smirnov (KS) test was used to check the shape of the oocyst levels' distribution. When the KS test indicated a nonnormal distribution, the rank of sum (Mann-Whitney) test was used to determine the statistical significance (Tables S5 and S6). For bacteria infection assays, 3–4 d after dsRNA injection, at least 50 of each control and experimental mosquitoes were injected with the same E. coli or S. aureus cultures. Dead mosquitoes were counted and removed daily for 7 d after bacterial challenge. Two-way analysis of variance was used to assess the significance of the gene-silencing effect on mosquito survival after challenge, with *p* < 0.05 deemed statistically significant. The RNAi gene-silencing assays were done as blinded tests with coded dsRNA samples. The effects on gene silencing are displayed in Table S6 as percentile of knockdown efficiency.

### Phylogenetic analysis.

Full-length or partial predicted sequences of MD2 homologues were aligned using the Clustal X program (ftp://ftp-igbmc.u-strasbg.fr/pub/ClustalX), and cladograms were constructed by neighbor-joining analysis and displayed through Treeview (http://darwin.zoology.gla.ac.uk/~rpage/treeviewx/download.html). AgMDL sequences were retrieved from Ensembl [53], and D. melanogaster MD2-like proteins (DmML) were retrieved from Flybase (http://flybase.bio.indiana.edu). DmMLs were named according to [21]. Genes were only considered as 1:1 orthologues if the relevant bootstrap values were higher than 800 (1,000 iterations).

## Supporting Information

Figure S1 Validation of Microarray-Assayed Gene Expression with Real-Time Quantitative RT-PCR The mean values for the expression data (log2 ratio) for 15 genes from three midgut assays (*P.f.* ookinete, *P.b.* ookinete, *P.f.* blood) obtained by microarray analysis were plotted against the corresponding mean expression values obtained with real-time RT-PCR from two biological replicates of each experiment. The Pearson correlation coefficient (*p* = 0.86), the best-fit linear-regression analysis (*R^2^* = 0.75), and the slope of the regression line (*m* = 0.996) demonstrated a high degree of correlation of the magnitude of regulation between the two assays. The individual values for all these genes are presented in Table S4.
(87 KB DOC)

Protocol S1 Click here for additional data file. Additional Information on Novel Immune Genes, Transcript Responses to *Plasmodium* Infection, and the Genes Selected for RNAi Screening
(414 KB DOC)

Table S1 Log2-Transformed Expression Ratios of Anopheles gambiae Genes Showing >1.74-Fold Regulation (0.8 in log2) under at Least One Experimental Condition in the Midgut Expression values of the following microarray assays are presented. Pf GUT, Pf wt gut/Pf CTRP^−^ gut; Pb GUT, Pb wt gut/Pb CTRP^−^ gut; Pf CTRP, Pf CTRP^−^ gut/blood-fed gut; Pf CARC, Pf wt carcass/Pf CTRP^−^ carcass; Pb CARC, Pb wt carcass/Pb CTRP^−^ carcass; Pf CTRPCARC, Pf CTRP^−^ carcass/blood-fed carcass.
(266 KB XLS)

Table S2 Log2-Transformed Expression Ratios of Anopheles gambiae Genes Showing >1.74-Fold Regulation (0.8 in log2) under at Least One Experimental Condition in Carcass Tissues Expression values of the following microarray assays are presented. Pf GUT, Pf wt gut/Pf CTRP^−^ gut; Pb GUT, Pb wt gut/Pb CTRP^−^ gut; Pf CTRP, Pf CTRP^−^ gut/blood-fed gut; Pf CARC, Pf wt carcass/Pf CTRP^−^ carcass; Pb CARC, Pb wt carcass/Pb CTRP^−^ carcass; Pf CTRPCARC, Pf CTRP^−^ carcass/blood-fed carcass.
(189 KB XLS)

Table S3 Primers Used to Produce PCR Amplicons for dsRNA Synthesis, Real-Time QRT-PCR for Microarray Validation, and Verification of Gene Silencing Underlined letters indicated the T7 promoter sequence. The same pair of forward and reverse primers was used for both dsRNA synthesis and QRT-PCR validation of microarray expression data. For the RT-PCR verification of gene silencing, the different veriF primers and reverse primers were used.
(80 KB DOC)

Table S4 Correlation of Microarray Expression Data with Real-Time QRT-PCR Comparison of the expression data from real-time quantitative RT-PCR (QRT) and DNA microarrays (Arrays) for 15 genes. For QRT-PCR, data were obtained from two biological and three technical replicates. The mean value for the regulation and standard error of the mean (SE) for the reactions were obtained from both QRT-PCR and array data. Pearson correlation (P) indicated the consistency between the two methods. N/A indicates the absence of microarray data.
(49 KB DOC)

Table S5 Effect of Gene Silencing on P. falciparum Infection (Oocyst Numbers) Plasmodium falciparum oocyst loads in midguts of gene knockdowns (KD) and their controls (GFP). The efficiency of gene KD (%) is presented in Table S6. The KD and GFP control mosquitoes in each dataset were fed on the same P. falciparum gametocyte culture. The results of equal numbers of midguts from all three experiments in each dataset were pooled. The total midgut numbers (midguts #), mean and standard error of oocyst numbers (Mean ± SE), range of oocyst numbers (range), *n-*fold difference of the mean oocyst numbers between gene KD and control (GFP) mosquitoes, and the *p* value from two independent probability tests (KS and Mann-Whitney test) are presented. Zero oocysts are also included for calculation of mean oocyst numbers. The repressive (−) effects of genes on parasite survival are shown in parentheses, with the asterisks indicating statistical significant at the 95% confidence level. NS indicates not significantly different. For calculation of mean oocyst numbers, midguts with zero oocysts were included.
(58 KB DOC)

Table S6 Effect of Gene Silencing on P. berghei Infection (Oocyst Numbers) P. berghei oocyst loads in midguts of gene knockdowns (KD) and their controls (GFP). The KD and GFP mosquitoes in each dataset were fed on the same infected mouse. Data represent a pool of at least three independent randomly selected experiments with equal numbers of midguts. The efficiency of gene KD (%) on average, the total midgut numbers (midguts #), mean, and standard error of oocyst numbers (Mean ± SE), range of oocyst numbers (range), *n-*fold difference of the mean oocyst numbers between gene KD and control (GFP) mosquitoes, and the *p* value from Kolmogorov-Smirnov test and Mann-Whitney test are presented. The repressive (−) effects of genes on parasite survival are shown in parentheses, with asterisks indicating the statistical significance at the 95% confidence level. NS indicates not significantly different. For calculation of mean oocyst numbers, midguts with zero oocysts were excluded.
(89 KB DOC)

Table S7 List of Selected ML Proteins for Phylogenetic Analysis (45 KB DOC)

## Abbreviations

AGBP: bacteria recognition family
AgMDL: Anopheles gambiae MD2-like protein
CTL: C-type lectin family
CTRP: circumsporozoite- and TRAP-related protein
dsRNA: double-strand RNA
FBN: fibrinogen domain immunolectin family
GALE: gal-lectin family
GNBP: Gram-negative bacteria binding protein family
KS: Kolmogorov-Smirnov
LRRD: leucine-rich repeat domain protein family
ML: MD2-like
PBS: phosphate-buffered saline
PGRP: peptidoglycan recognition protein family
Tep: thioester-containing protein family
wt: wild-type
