# Supplementary material for: Anopheles gambiae Immune Responses to Human and Rodent Plasmodium Parasite Species
Source: PLoS Pathog. 2006 Jun 9;2(6):e52. doi: 10.1371/journal.ppat.0020052 (PMC1475661; doi:10.1371/journal.ppat.0020052)
Supplement: Supplementary file 1 [file ppat.0020052.sd001.doc]

**Supporting Information**

Note: Gene IDs have been abbreviated by omitting the “NSANGT000000” part.

**Novel mosquito immune genes**

Several novel infection-responsive transcripts encoding proteins that have not been linked with *A. gambiae* responses to *Plasmodium* infection previously but are homologues of innate immune genes of other organisms were identified. Five transcripts encoding vertebrate MD2 protein homologues are induced under different conditions of infection. MD2 serves as a co-receptor for LPS, together with Toll-like receptor 4, and is necessary for the activation of the Toll pathway (discussed in detail below) [15]. As many as 24 leucine-rich repeat (LRR) domain-containing protein transcripts, LRRDs, were induced upon challenge. LRR domains are common in several types of immune proteins, including the Toll receptors (described below) [16,17]. The homologue of a pacifastin-like serine protease inhibitor (E18532 in group 1) has been shown to inhibit the endogenous proteolytic activating cascade of prophenoloxidases (Table 1) [13]. The mouse homologue of the F-spondin transcripts, spondin 2 (E8856 in group 1 and E19994 in group7(1)), is a pattern recognition receptor implicated in antibacterial defense [1]. An apolipoprotein D, APOD (E28106 in group 1), has been linked with both anti-*Plasmodium* and antimicrobial defense in *A. gambiae* (discussed below). Apolipoproteins have been shown to function as effectors in the fish innate immune system, and apolipophorins are lipopolysaccharide-binding proteins of the innate immune system in the moth [*Galleria mellonella*](http://linus.socs.uts.edu.au/~don/larvae/pyra/mellon.html). Other insect apolipoproteins have been associated with pattern recognition, encapsulation, and antimicrobial peptide production. [2-4]. One induced transcript encodes a transferrin (E21949 in group 1); such proteins are believed to inhibit pathogen growth by iron sequestration [5]. Several cuticle proteins are induced by infection in both midgut and carcass tissues. These proteins have adhesive domains, are essential for wound healing, and have previously been implicated in the response to microbial challenge [6,7]. HECY1 and HECY2 (E22058 and E22055 in group 4) were both induced in the carcass upon *P. falciparum* ookinete invasion and belong to the hemocyanin gene family. Hemocyanins are copper-containing oxygen transport proteins found in the hemolymph of many invertebrates. They exhibit prophenoloxidase activity and may therefore play a role in melanization as well as in the production of antimicrobial substances [8]. A Kazal-type serine protease inhibitor transcript, *SPI-KAZ* (E19065 in group 7(1)), was specifically induced in the gut by *P. bergehi* ookinete invasion. Kazal inhibitors, which contain between 1 and 7 Kazal-type inhibitor repeats, can inhibit a number of immunity-related serine proteases, such as trypsins and elastases [14]. The Saposin-like protein transcript (E13093 in group 7(2)) is homologous to Saposin B, which has been implicated in granule-mediated lysis of pathogens [9]. One of the major killing mechanisms of *Plasmodium* in the midgut epithelium has a lytic phenotype, and granules have been observed in the proximity of dying ookinetes [10-12].

**Other mosquito responses to ookinete invasion and malaria-infected blood**

Analyses of gene expression responses to infection can provide a wealth of information on how a variety biological processes are affected in the mosquito. Invasion of the midgut epithelium by *P. falciparum* and *P. berghei* ookinetes regulated 3% and 7.5% of the predicted mosquito transcriptome, respectively, in the midgut and carcass tissues. Infected blood alone stimulated the regulation of 13% of all mosquito genes. Known immune proteins were only encoded by a minority of these genes, which mostly represented components involved in nucleic acid and protein metabolism, energy metabolism, and stress responses. Expression of genes encoding a variety of structural and cytoskeletal components were affected by *Plasmodium* infection, as a result of the midgut invasion itself, and most likely also of the subsequent expulsion of the invaded apoptotic cells into the midgut lumen [18-20]. Gene expression signatures associated with such processes are prominent in the responses to *P. berghei* ookinete invasion, which involved the induction of 21 putative cytoskeletal and structural genes encoding actins and actin-binding proteins, microtubule components, basal membrane components, and a variety of other proteins (supplementary data table S1, 2). With the exception of the gene encoding an actin (E22306), these genes were not seen to be induced in the *P. falciparum* ookinete-invaded midguts, although a recent study [18] has confirmed a similar ookinete invasion mechanism and midgut epithelial response at the cellular level. Several cytoskeletal protein genes were also expressed at higher levels in mosquitoes that had ingested infected blood than in mosquitoes fed on healthy blood. This difference may be related to ingestion of a larger volume when feeding on the less viscous infected blood. *P. berghei* ookinete invasion induced 28 genes that are predicted to be linked with mitochondrial, oxidoreductive and stress-related processes, in contrast to only 14 genes in *P. falciparum*-invaded midguts.

These differences in gene expression between *P. falciparum* and *P. berghei* ookinete-invaded guts are most likely the result of the much larger number of invading *P. berghei* ookinetes. It is noteworthy that infection with *P. berghei* frequently results in a >30% mosquito mortality during and after the midgut is invaded, while the mortality of *P. falciparum*-infected mosquitoes rarely exceeds 10% (Dimopoulos, unpublished observations). Gene expression in the small number of cells that are being invaded can be masked and diluted by the constitutive expression levels of these genes in non-invaded cells. Transcriptional induction of cytoskeletal components by *P. berghei* infection has also been documented in the *A. stephensi* mosquito species [18]. Several genes encoding putative apoptosis factors, such as cathepsins and caspases, were induced under different conditions of *Plasmodium* infection (supplemental data S1, 2). These expression signatures are most likely related to the death of invaded cells and immune responses [19-21]. Induction of genes encoding putative blood-digesting enzymes, such as trypsins and peptidases, upon *P. berghei* ookinete invasion may indicate interactions between the mosquito’s infection responsive and digestive machinery (supplemental data S1, 2). Several trypsins have previously been shown to be regulated upon bacterial challenge in *A. gambiae* [6]. Several genes encoding chemosensory-related factors such as odorant-binding proteins, olfactory receptors, and arrestins, are induced under various experimental conditions and may indicate involvement of the chemosensory system in sensing infection in the blood, and not only detection and discrimination of odorants in the mosquito’s external environment (supplemental material table S1, 2) [22,23].

**Genes selected for RNAi screening.**

Among the tested genes was *AgMDL1* (E20083), which encodes an MD-2-like protein that belongs to a 13-member *A. gambiae* gene family*.* MD-2 is essential for the TLR4-mediated immune response of mammalian cells to LPS. TLR4 interaction with LPS requires the formation of a receptor complex with MD-2, which is dependent on the Cys95 and Cys105 residues that are conserved in the *A. gambiae* AgMDL1. The MD-2-like (ML) lipid recognition domain found in AgMDL1 is present in a variety of proteins with diverse functions; there are at least 155 ML family members (Figure 4). Other invertebrate members of this family include seven mite allergen proteins, eight *D. melanogaster* proteins, and five *C. elegans* proteins [24,25]. Three fibrinogen domain-like immunolectins, FBN8 (E11252), FBN9 (E11248), and FBN39 (E21380), belong to a large *A. gambiae* gene family comprising over 50 members*.* The *D. melanogaste*r genome has only13 FBN genes, suggesting that the *A. gambiae* FBN family may possess essential mosquito-specific functional attributes [26]. Characterization of the FBN gene family has suggested that the FBNs have structural features that allow them to recognize parasites and play an essential role in the mosquito’s innate immunity, in addition to the physiological processes associated with blood feeding [27]. *Tep4* (E21216) belongs to the *A. gambiae* 15-member thioester-containing protein gene family (Tep) that includes *Tep1,* a phagocytic and anti-*Plasmodium* factor. Several Tep genes are induced by infection, but whether these *Tep4, Tep6, Tep12* and *Tep14* genes have functions similar to those of *Tep1* is as yet unknown [11]. SP-CLIP1 (E20158) encodes a clip domain serine protease that may be implicated in immune signal amplification cascades that can activate a variety of defense reactions. These serine protease cascades are regulated by serine protease inhibitors [28,29]. HECY1 (E22058) belongs to the hemocyanin family of proteins that exhibit prophenoloxidase activity and may therefore play a role in melanization reactions as well as in the production of antimicrobial substances [8]. Four novel infection responsive genes, *IRSP1* (E21028)*, IRSP2* (E19220)*, IRSP3* (E09630) and *IRSP5* (E19451), encode short secreted peptides that may represent novel antimicrobial factors or other soluble immune factors. These genes were strongly induced by *P. falciparum* ookinete invasion of the midgut (Tables 1, S1 and S2; Figures 2 and 3). In contrast, *IRSP3* did not exhibit any effect on infection and is therefore not included in Table 1 or Figures 2 or 3. APOD (E28106) encodes an apolipoprotein that has been linked to immunity in several other organisms (discussed above). Gambicin (E13255) is a 60-amino acid antimicrobial peptide that is also present in the *Aedes aegypti* genome but not that of *D. melanogaster;* it may therefore represent a mosquito-specific immune factor. Gambicin has been shown to possess anti-*P. berghei* activity *in vivo* and *in vitro* and is active against fungi and bacteria [30]. *LRRD7* (E21822) is a leucine-rich repeat domain (LRR)-containing protein that belongs to the broader gene family ENANGSF00000000444, which also includes several *A. gambiae* Toll receptor genes. The LRR domain protein LRIM1 (E13041) was recently established as a major anti-*Plasmodium* factor [12]. *GNBPB1* (E17694) belongs to the gram-negative bacteria-binding protein gene family, which has six members. GNBP has been implicated in signaling cascades that activate the Toll pathway in *D.* melanogaster [31]. *PGRP-amidase* (E12978) encodes an amidase-like peptidoglycan recognition protein. In mammals, these PGRPs have antibacterial activity, while PGRP-S and PGRP-Sa in *D. melanogaster* have been known to function as pattern recognition receptors [32,33]. Several novel genes with unknown function but with sequences characteristic of antimicrobial peptides were also included in the analysis.

**References**

1. He YW, Li H, Zhang J, Hsu CL, Lin E, et al. (2004) The extracellular matrix protein mindin is a pattern-recognition molecule for microbial pathogens. Nat Immunol 5: 88-97.

2. Shi L, Paskewitz SM (2004) Identification and molecular characterization of two immune-responsive chitinase-like proteins from Anopheles gambiae. Insect Mol Biol 13: 387-398.

3. Pratt CC, Weers PM (2004) Lipopolysaccharide binding of an exchangeable apolipoprotein, apolipophorin III, from Galleria mellonella. Biol Chem 385: 1113-1119.

4. Whitten MM, Tew IF, Lee BL, Ratcliffe NA (2004) A novel role for an insect apolipoprotein (apolipophorin III) in beta-1,3-glucan pattern recognition and cellular encapsulation reactions. J Immunol 172: 2177-2185.

5. Harizanova N, Georgieva T, Dunkov BC, Yoshiga T, Law JH (2005) Aedes aegypti transferrin. Gene structure, expression pattern, and regulation. Insect Mol Biol 14: 79-88.

6. Aguilar R, Jedlicka AE, Mintz M, Mahairaki V, Scott AL, et al. (2005) Global gene expression analysis of Anopheles gambiae responses to microbial challenge. Insect Biochem Mol Biol 35: 709-719.

7. Galko MJ, Krasnow MA (2004) Cellular and genetic analysis of wound healing in Drosophila larvae. PLoS Biol 2: E239.

8. Cerenius L, Soderhall K (2004) The prophenoloxidase-activating system in invertebrates. Immunol Rev 198: 116-126.

9. Ernst WA, Thoma-Uszynski S, Teitelbaum R, Ko C, Hanson DA, et al. (2000) Granulysin, a T cell product, kills bacteria by altering membrane permeability. J Immunol 165: 7102-7108.

10. Vernick KD, Fujioka H, Seeley DC, Tandler B, Aikawa M, et al. (1995) Plasmodium gallinaceum: a refractory mechanism of ookinete killing in the mosquito, Anopheles gambiae. Exp Parasitol 80: 583-595.

11. Blandin S, Levashina EA (2004) Mosquito immune responses against malaria parasites. Curr Opin Immunol 16: 16-20.

12. Osta MA, Christophides GK, Kafatos FC (2004) Effects of mosquito genes on Plasmodium development. Science 303: 2030-2032.

13. Simonet G, Claeys I, Franssens V, De Loof A, Broeck JV (2003) Genomics, evolution and biological functions of the pacifastin peptide family: a conserved serine protease inhibitor family in arthropods. Peptides 24: 1633-1644.

14. Sommerhoff CP, Sollner C, Mentele R, Piechottka GP, Auerswald EA, et al. (1994) A Kazal-type inhibitor of human mast cell tryptase: isolation from the medical leech Hirudo medicinalis, characterization, and sequence analysis. Biol Chem Hoppe Seyler 375: 685-694.

15. Miller SI, Ernst RK, Bader MW (2005) LPS, TLR4 and infectious disease diversity. Nat Rev Microbiol 3: 36-46.

16. Anderson KV (2000) Toll signaling pathways in the innate immune response. Curr Opin Immunol 12: 13-19.

17. Kobe B, Deisenhofer J (1994) The leucine-rich repeat: a versatile binding motif. Trends Biochem Sci 19: 415-421.

18. Xu X, Dong Y, Abraham EG, Kocan A, Srinivasan P, et al. (2005) Transcriptome analysis of Anopheles stephensi-Plasmodium berghei interactions. Mol Biochem Parasitol 142: 76-87.

19. Vlachou D, Schlegelmilch T, Christophides GK, Kafatos FC (2005) Functional genomic analysis of midgut epithelial responses in Anopheles during Plasmodium invasion. Curr Biol 15: 1185-1195.

20. Han YS, Thompson J, Kafatos FC, Barillas-Mury C (2000) Molecular interactions between Anopheles stephensi midgut cells and Plasmodium berghei: the time bomb theory of ookinete invasion of mosquitoes. Embo J 19: 6030-6040.

21. Vlachou D, Zimmermann T, Cantera R, Janse CJ, Waters AP, et al. (2004) Real-time, in vivo analysis of malaria ookinete locomotion and mosquito midgut invasion. Cell Microbiol 6: 671-685.

22. Biessmann H, Walter MF, Dimitratos S, Woods D (2002) Isolation of cDNA clones encoding putative odourant binding proteins from the antennae of the malaria-transmitting mosquito, Anopheles gambiae. Insect Mol Biol 11: 123-132.

23. Melo AC, Rutzler M, Pitts RJ, Zwiebel LJ (2004) Identification of a chemosensory receptor from the yellow fever mosquito, Aedes aegypti, that is highly conserved and expressed in olfactory and gustatory organs. Chem Senses 29: 403-410.

24. Inohara N, Nunez G (2002) ML -- a conserved domain involved in innate immunity and lipid metabolism. Trends Biochem Sci 27: 219-221.

25. Nagai Y, Akashi S, Nagafuku M, Ogata M, Iwakura Y, et al. (2002) Essential role of MD-2 in LPS responsiveness and TLR4 distribution. Nat Immunol 3: 667-672.

26. Zdobnov EM, von Mering C, Letunic I, Torrents D, Suyama M, et al. (2002) Comparative genome and proteome analysis of Anopheles gambiae and Drosophila melanogaster. Science 298: 149-159.

27. Wang X, Rocheleau TA, Fuchs JF, Hillyer JF, Chen CC, et al. (2004) A novel lectin with a fibrinogen-like domain and its potential involvement in the innate immune response of Armigeres subalbatus against bacteria. Insect Mol Biol 13: 273-282.

28. Gorman MJ, Paskewitz SM (2001) Serine proteases as mediators of mosquito immune responses. Insect Biochem Mol Biol 31: 257-262.

29. Dimopoulos G (2003) Insect immunity and its implication in mosquito-malaria interactions. Cell Microbiol 5: 3-14.

30. Vizioli J, Bulet P, Hoffmann JA, Kafatos FC, Muller HM, et al. (2001) Gambicin: a novel immune responsive antimicrobial peptide from the malaria vector Anopheles gambiae. Proc Natl Acad Sci U S A 98: 12630-12635.

31. Gobert V, Gottar M, Matskevich AA, Rutschmann S, Royet J, et al. (2003) Dual activation of the Drosophila toll pathway by two pattern recognition receptors. Science 302: 2126-2130.

32. Gottar M, Gobert V, Michel T, Belvin M, Duyk G, et al. (2002) The Drosophila immune response against Gram-negative bacteria is mediated by a peptidoglycan recognition protein. Nature 416: 640-644.

33. Bischoff V, Vignal C, Boneca IG, Michel T, Hoffmann JA, et al. (2004) Function of the drosophila pattern-recognition receptor PGRP-SD in the detection of Gram-positive bacteria. Nat Immunol 5: 1175-1180.

34. Blandin S, Moita LF, Kocher T, Wilm M, Kafatos FC, et al. (2002) Reverse genetics in the mosquito Anopheles gambiae: targeted disruption of the Defensin gene. EMBO Rep 3: 852-856.

**Tables S1 and S2** (attached as separate files). Log2-transformed expression ratios of *Anopheles gambiae* genes showing >1.74-fold regulation (0.8 in log2) under at least one experimental condition in the midgut (S1) and carcass tissues (S2). Expression values of the following microarray assays are presented. Pf GUT: Pf wt gut/Pf CTRP^-^ gut, Pb GUT: Pb wt gut/Pb CTRP^-^ gut, Pf CTRP: Pf CTRP^-^ gut /blood-fed gut, Pf CARC. Pf wt carcass/Pf CTRP^-^ carcass, Pb CARC. Pb wt carcass/Pb CTRP^-^ carcass, Pf CTRPCARC. Pf CTRP^-^ carcass/blood-fed carcass.

**Table S3.** Primers used to produce PCR amplicons for dsRNA synthesis, real-time QRT-PCR for microarray validation, and verification of gene silencing.

| Primer Name | Gene ID | ENSANGT# (last 5 digits) | Primer sequence |
| --- | --- | --- | --- |
| *Tep1*-F | E14368 | 16857 | 5’- TAATACGACTCACTATAGGTTTGTGGGCCTTAAAGCGCTG -3’ |
| *Tep1*-R |  |  | 5’- TAATACGACTCACTATAGGACCACGTAACCGCTCGGTAAG-3’ |
| *Tep1-*veriF |  |  | 5’- GGTGAATCAACGGTACGTTA -3’ |
| *AgMDL1*-F | E17594 | 20083 | 5’- TAATACGACTCACTATAGGTTCGATTTTACCCCCGAGT-3’ |
| *AgMDL1*-R |  |  | 5’- TAATACGACTCACTATAGGTCCTAAGTGCGCTTGCTCT-3’ |
| *AgMDL1-*veriF |  |  | 5' - GTACAATCCATGAGGTTTCGATC - 3' |
| *LRRD7*-F | [E19333](http://www.ensembl.org/Anopheles_gambiae/geneview?gene=ENSANGG00000019333&db=core) | 21822 | 5’- TAATACGACTCACTATAGTCGGTGAGCAACAGTTTGAC-3’ |
| *LRRD7-*R |  |  | 5’- TAATACGACTCACTATAGCTTCATTCCCGCTAATGCTC-3’ |
| *LRRD7*-veriF |  |  | 5' - CGCCACGATCGAAAGCACCGCGT - 3' |
| *SPCLIP1*-F | [E17669](http://www.ensembl.org/Anopheles_gambiae/geneview?gene=ENSANGG00000017669&db=core) | 20158 | 5’- TAATACGACTCACTATAGGTCACCGAACACGTCCAAC-3’ |
| *SPCLIP1*-R |  |  | 5’- TAATACGACTCACTATAGCTGCATGGCCCTACGTCTA-3’ |
| *SPCLIP-*veriF |  |  | 5' - GCCGTAGAGGAGCTTATCATCCA - 3' |
| *IRSP2*-F | [E1673](http://www.ensembl.org/Anopheles_gambiae/geneview?gene=ENSANGG00000016731&db=core)1 | 19220 | 5’- TAATACGACTCACTATAGGCTAGCGTTGTCGTTTGCTC-3’ |
| *IRSP2*-R |  |  | 5’- TAATACGACTCACTATAGTGACATTTCAATCCCATTCG-3’ |
| *IRSP2*-veriF |  |  | 5’- CTAATGCGCTCGGTGCGGGCAAT -3’ |
| *IRSP1*-F | [E18539](http://www.ensembl.org/Anopheles_gambiae/geneview?gene=ENSANGG00000018539&db=core) | 21028 | 5’-TAATACGACTCACTATAGCTGGAAGGATGGACAGATGG-3’ |
| *IRSP1*-R |  |  | 5’- TAATACGACTCACTATAGGGATTGAAACGGATCAAGG-3’ |
| *IRSP1-*veriF |  |  | 5' - CCGATCGGACACTTCACCCAGAT - 3' |
| *IRSP5*-F | E16962 | 19451 | 5’- TAATACGACTCACTATAGCAGTTTGCTGTCTGCTGCTC |
| *IRSP5*-R |  |  | 5’- TAATACGACTCACTATAGTCGCTCTCAATCTTCCAGTG |
| *IRSP5-*veriF |  |  | 5' - GATATCGATTGCTGGTACTGTTG - 3' |
| *PGRP-AMIDASE*-F | E10489 | 12978 | 5’- TAATACGACTCACTATAGAGGATAGGCGGCAGTGAAC-3’ |
| *PGRP-AMIDASE* -R |  |  | 5’- TAATACGACTCACTATAGTCCATGTGGAAGGACTGGA-3’ |
| *PGRP-AMIDASE -*veriF |  |  | 5’- CGATAAGTAATCGACAATTCT - 3' |
| *Gambicin*-F | E10766 | 13255 | 5’- TAATACGACTCACTATAGGGCTATCTCAACCGGAAGG-3’ |
| *Gambicin*-R |  |  | 5’- TAATACGACTCACTATAGCCAACGTCTGGCACTGATT-3’ |
| *Gambicin-*veriF |  |  | 5' - CGGCAGCCGTTGCGGATGCAATG - 3' |
| *Tep4*-F | [E18727](http://www.ensembl.org/Anopheles_gambiae/geneview?gene=ENSANGG00000018727&db=core) | 21216 | 5’- TAATACGACTCACTATAGTGTTGATTGCACTGCTGGA-3’ |
| *Tep4*-R |  |  | 5’- TAATACGACTCACTATAGGAGGTCCCGTCCACGTACT -3’ |
| *Tep4-*veriF |  |  | 5' - GGAGGGGCCGTCTTTCTGACCGC - 3' |
| *AgBP1*-F | [E05241](http://www.ensembl.org/Anopheles_gambiae/geneview?gene=ENSANGG00000005241&db=core) | 06959 | 5’- TAATACGACTCACTATAGCATTCCCCTGCTGAAGGATA-3’ |
| *AgBP1*-R |  |  | 5’- TAATACGACTCACTATAGATCTTGCGCAACGGATACTC-3’ |
| *AgBP-*veriF |  |  | 5’- GTTCACCGGTGACAGCGTGCTC -3’ |
| *APOD*-F | E16962 | 28106 | 5’- TAATACGACTCACTATAGTAGCTATCCGGACGTGTTCC-3’ |
| *APOD*-R |  |  | 5’- TAATACGACTCACTATAGTTGCATTTTGCCTGACTTTG-3’ |
| *APOD-*veriF |  |  | 5' - CGAGAAGGACTGTGACTGCGGGT - 3' |
| *HECY1*-F | [E19569](http://www.ensembl.org/Anopheles_gambiae/geneview?gene=ENSANGG00000019569&db=core) | 22058 | 5’- TAATACGACTCACTATAGTCGAACGATCTCGGTACGATT -3’ |
| *HECY1*-R |  |  | 5’- TAATACGACTCACTATAGTCCGTTCGATTGGATCAGATT -3’ |
| *HECY1-*veriF |  |  | 5' - GTGCGTTCATCCCGAGCAACTAC - 3' |
| *KIN*-F | E11642 | 14131 | 5’- TAATACGACTCACTATAGTTTGTTGGCGATCTTTGTCA -3’ |
| *KIN*-R |  |  | 5’- TAATACGACTCACTATAGGCATGATCACATTTCCCCTAGT -3’ |
| *KIN-*veriF |  |  | 5' - CTGACCAGTCCGCCCGGAGACGC - 3' |
| *FBN9-F* | E08759 | 11248 | 5’- TAATACGACTCACTATAGCCAAGATGTCGGGCAAGTAT -3’ |
| *FBN9-R* |  |  | 5’- TAATACGACTCACTATAGTTGTGGTACGTCAGCGAGTC -3’ |
| *FBN9-*veriF |  |  | 5’- CGCTCCCTGTTCGAGCTGCA -3’ |
| *FBN8-F* | E08763 | 11252 | 5’- TAATACGACTCACTATAGTTCAAGGGCAATTTTTGGTC -3’ |
| *FBN8-R* |  |  | 5’- TAATACGACTCACTATAGATATTGTTCCGCCTCACTGC -3’ |
| *FBN8-*veriF |  |  | 5’- CCACGAACAAATGCGGAACG -3’ |
| *FBN39-F* | E18891 | 21380 | 5’- TAATACGACTCACTATAGCGCTACACCCTGTTCGAGAT -3’ |
| *FBN39-R* |  |  | 5’- TAATACGACTCACTATAGCCGTACTGGTCGTGGTCCTG -3’ |
| *FBN39-*veriF |  |  | 5’-GGCCGAATCGTTCAAGGGCG -3’ |
| *GNBPB1-F* | E15205 | 17694 | 5’- TAATACGACTCACTATAGGTTCGGGTTCGTGTGGAC -3’ |
| *GNBPB1-R* |  |  | 5’- TAATACGACTCACTATAG ACCAGCAACGAGGCAGAC -3’ |
| *GNBPB1-*veriF |  |  | 5’- ACGGCAACGCTAACGAAG -3’ |
| *IRSP3-F* | [E22382](http://www.ensembl.org/Anopheles_gambiae/geneview?gene=ENSANGG00000022382&db=core) | 09630 | 5’- TAATACGACTCACTATAGAGTCGTTCAACCAGGGCTTC -3’ |
| *IRSP3-R* |  |  | 5’- TAATACGACTCACTATAGCCAGTTTCGAGGCAGAGGTA -3’ |
| Underlined letters indicated the T7 promoter sequence. The same pair of forward and reverse primers was used for both dsRNA synthesis and QT-RT PCR validation of microarray expression data. For the RT-PCR verification of gene silencing, the different veriF primers and reverse primers were used. | | | |

**Table S4.** Correlation of microarray expression data with real-time QRT-PCR

|  | ***P.f.* ookinete (gut)** | |  | ***P.b.* ookinete (gut)** | |  | ***P.f.* blood (gut)** | |  |  |
| --- | --- | --- | --- | --- | --- | --- | --- | --- | --- | --- |
|  | QRT ± SE | Arrays ± SE |  | QRT ± SE | Arrays ± SE |  | QRT ± SE | Arrays ± SE |  | P |
| *Tep1* | 2.30 ± 0.14 | 2.26 ± 0.09 |  | 0.82 ± 0.09 | -0.30 ± 0.01 |  | -0.28 ± 0.09 | 0.14 ± 0.10 |  | 0.83 |
| *AgMDL1* | 1.43 ± 0.02 | 1.57 ± 0.21 |  | -1.08 ± 0.08 | -0. 09 ± 0.23 | | -0.44 ± 0.11 | -0.87 ± 0.08 |  | 0.84 |
| *SPCLIP1* | 1.51 ± 0.02 | 1.80 ± 0.07 |  | -0.33 ± 0.06 | -0.32 ± 0.07 |  | -0.33 ± 0.13 | -0.06 ± 0.06 |  | 0.99 |
| *LRRD7* | 2.23 ± 0.12 | 2.18 ± 0.25 |  | -1.87 ± 0.03 | -1.62 ± 0.15 |  | -1.56 ± 0.10 | 1.02 ± 0.11 |  | 0.78 |
| *APOD* | 0.91 ± 0.08 | 1.30 ± 0.12 |  | -1.99 ± 0.10 | -0.89 ± 0.03 |  | 0.43 ± 0.19 | -0.07 ± 0.01 |  | 0.87 |
| *IRSP1* | 2.34 ± 0.22 | 2.33 ± 0.35 |  | -0.7 ± 0.18 | -0.06 ± 0.12 |  | N/A | N/A |  | N/A |
| *IRSP2* | 2.00 ± 0.05 | 2.24 ± 0.23 |  | -0.99 ± 0.12 | -0.44 ± 0.03 |  | -2.69 ± 0.09 | -2.20 ± 0.26 |  | 0.99 |
| *GAMBICIN* | 0.02 ± 0.26 | -0.30 ± 0.05 | | 0.24 ± 0.11 | 0.06 ± 0.26 |  | 3.53 ± 0.20 | 3.15 ± 0.36 |  | 0.90 |
| *IRSP5* | 2.45 ± 0.05 | 2.90 ± 0.26 |  | -2.14 ± 0.04 | -2.10 ± 0.10 |  | N/A | N/A |  | N/A |
| *TEP4* | 2.41 ± 0.06 | 1.66 ± 0.12 |  | 0.56 ± 0.02 | -0.17 ± 0.10 |  | -0.026 ± 0.05 | 0.03 ± 0.02 |  | 0.89 |
| *KIN* | 1.45 ± 0.11 | 1.77 ± 0.13 |  | -1.61 ± 0.10 | -0.49 ± 0.12 |  | 2.16 ± 0.02 | 1.16 ± 0.03 |  | 0.90 |
| *PGRP-AMIDASE* | 0.90 ± 0.10 | -0.02 ± 0.14 | | 1.03 ± 0.13 | 1.64 ± 0.28 |  | 2.15 ± 0.28 | 1.40 ± 0.09 |  | 0.93 |
| *AgBP1* | 1.87 ± 0.17 | 1.78 ± 0.17 |  | -0.13 ± 0.03 | -0.99 ± 0.01 |  | -0.48 ± 0.13 | -0.76 ± 0.09 |  | 0.98 |
| *HECY1* | 2.32 ± 0.07 | 1.54 ± 0.15 |  | -3.57 ± 0.23 | N/A |  | -0.96 ± 0.20 | -0.73 ± 0.06 |  | N/A |
| *IRSP6* | 1.97 ± 0.05 | 2.31 ± 0.06 |  | -0.13 ± 0.04 | 0.02 ± 0.05 |  | 3.23 ± 0.05 | 1.36 ± 0.16 |  | 0.79 |

Comparison of the expression data from real-time quantitative RT-PCR (QRT) and DNA microarrays (Arrays) for 15 genes. For QRT-PCR, data were obtained from two biological and three technical replicates. The mean value for the regulation and standard error of the mean (SE) for the reactions were obtained from both QRT-PCR and array data. Pearson correlation (P) indicated the consistency between the two methods. N/A indicates the absence of microarray data.

**Figure S1.** Validation of microarray-assayed gene expression with real-time quantitative RT-PCR. The mean values for the expression data (log2 ratio) for 15 genes from three midgut assays (*P.f.* ookinete, *P.b.* ookinete, *P.f.* blood) obtained by microarray analysis were plotted against the corresponding mean expression values obtained with real-time RT-PCR from two biological replicates of each experiment. The Pearson correlation coefficient (p =0.86), the best-fit linear-regression analysis (R^2^ = 0.75), and the slope of the regression line (m = 0.996) demonstrated a high degree of correlation of the magnitude of regulation between the two assays. The individual values for all these genes are presented in Table S4.

**Table S5.** Effect of gene silencing on *P. falciparum* infection (oocyst numbers)

| Gene | Midguts # | Mean Oocysts ± SE | Oocysts(range) | p value KSTest | p value Mann-Whitney | *n*-Fold Difference | Significance |  | | | | | |
| --- | --- | --- | --- | --- | --- | --- | --- | --- | --- | --- | --- | --- | --- |
| *GFP* | 54 | 1.12 ± 0.36 | 0-6 | 0.00 |  |  |  |  | | | | | |
| *Tep1* | 48 | 2.32 ± 0.49 | 0-14 | 0.01 | 0.026 | 2.06 | (-)* |  | | | | | |
| *GFP* | 48 | 1.96 ± 0.24 | 0-9 |  |  |  |  |  | | | | | |
| *AgMDL1* | 75 | 4.06 ± 0.61 | 0-22 | 0.05 | 0.005 | 2.07 | (-)* |  | | | | | |
| *GFP* | 51 | 1.28 ± 0.22 | 0-5 |  |  |  |  |  | | | | | |
| *FBN39* | 63 | 2.02 ± 0.37 | 0-7 | 0.01 | 0.049 | 1.58 | (-)* |  | | | | | |
| *GFP* | 75 | 1.13 ± 0.19 | 0-6 |  |  |  |  |  | | | | | |
| *SPCLIP1* | 93 | 1.98 ± 0.36 | 0-12 | 0.00 | 0.011 | 1.76 | (-)* |  | | | | | |
| *GFP* | 84 | 1.26 ± 0.17 | 0-6 |  |  |  |  |  | | | | | |
| *LRRD7* | 114 | 2.24 ± 0.49 | 0-27 | 0.03 | 0.010 | 1.78 | (-)* |  | | | | | |
| *GFP* | 39 | 4.70 ± 0.46 | 0-9 |  |  |  |  |  | | | | | |
| *APOD* | 63 | 11.00 ± 0.60 | 0-63 | 0.00 | 0.003 | 2.34 | (-)* |  | | | | | |
| *GFP* | 45 | 2.36 ± 0.43 | 0-9 |  |  |  |  |  | | | | | |
| *IRSP1* | 54 | 4.88 ± 0.62 | 0-51 | 0.01 | 0.045 | 2.07 | (-)* |  | | | | | |
| *GFP* | 63 | 1.26 ± 0.36 | 0-6 |  |  |  |  |  | | | | | |
| *IRSP2* | 72 | 1.77 ± 0.37 | 0-24 | 0.01 | 0.233 | 1.41 | NS |  | | | | | |
| *GFP* | 51 | 1.28 ± 0.42 | 0-5 |  |  |  |  |  | | | | | |
| *FBN9* | 60 | 2.18 ± 0.32 | 0-6 | 0.02 | 0.018 | 1.70 | (-)* |  | | | | | |
| *GFP* | 51 | 1.28 ± 0.20 | 0-5 |  |  |  |  |  | | | | | |
| *FBN8* | 63 | 2.02 ± 0.32 | 0-7 | 0.01 | 0.045 | 1.58 | (-)* |  | | | | | |
| *GFP* | 45 | 2.36 ± 0.21 | 0-9 |  |  |  |  |  | | | | | |
| *GAMBICIN* | 66 | 2.67 ± 0.38 | 0-20 | 0.01 | 0.245 | 1.13 | NS |  | | | | | |
| *GFP* | 45 | 2.36 ± 0.21 | 0-9 |  |  |  |  |  | | | | | |
| *IRSP5* | 54 | 2.07 ± 0.39 | 0-29 | 0.01 | 0.356 | -1.14 | NS |  | | | | | |
| *Plasmodium* *falciparum* oocyst loads in midguts of gene knockdowns (KD) and their controls (GFP). The efficiency of gene KD (%) is presented in Table S6. The KD and GFP control mosquitoes in each dataset were fed on the same *P. falciparum* gametocyte culture. The results of equal numbers of midguts from all three experiments in each dataset were pooled. The total midgut numbers (midguts #), mean and standard error of oocyst numbers (Mean±SE), range of oocyst numbers (range), *n-*fold difference of the mean oocyst numbers between gene KD and control (GFP) mosquitoes, and the p-value from two independent probability tests (Kolmogorov-Smirnov (KS) and Mann-Whitney test*)* are presented. Zero oocysts are also included for calculation of mean oocyst numbers. The repressive (-) effects of genes on parasite survival are shown in parentheses, with the asterisks indicating statistical significant at the 95% confidence level. NS indicates not significantly different. For calculation of mean oocyst numbers, midguts with zero oocysts were included. | | | | | | | | |  |  |  |  |  |

**Table S6.** Effect of gene silencing on *P. berghei* infection (oocyst numbers)

| Gene | KD% (± SE) | Midguts # | Mean Oocysts ± SE | Oocysts(range) | p value KS-Test | p value Mann-Whitney | *n*-Fold Difference | Signif icance | |  | | | | | |
| --- | --- | --- | --- | --- | --- | --- | --- | --- | --- | --- | --- | --- | --- | --- | --- |
| *GFP* |  | 42 | 93.0 ± 12.8 | 1-296 | 0.00 |  | 1.00 |  | |  | | | | | |
| *Tep1* | 78 (±9) | 42 | 205.6 ± 21.7 | 1-500 | 0.00 | 0.000 | 2.21 | (-)* | |  | | | | | |
| *GFP* |  | 45 | 87.2 ± 8.3 | 1-198 |  |  |  |  | |  | | | | | |
| *AgMDL1* | 66 (±8) | 78 | 60.2 ± 4.8 | 1-185 | 0.01 | 0.051 | -1.49 | NS | |  | | | | | |
| *GFP* |  | 39 | 70.0 ± 10.0 | 1-189 |  |  |  |  | |  | | | | | |
| *FBN39* | 58 (±10) | 57 | 84.8 ± 7.6 | 1-213 | 0.03 | 0.374 | 1.17 | NS | |  | | | | | |
| *GFP* |  | 36 | 82.0 ± 14.7 | 1-320 |  |  |  |  | |  | | | | | |
| *SPCLIP1* | 88 (±8) | 48 | 152.7 ± 14.0 | 1-430 | 0.00 | 0.001 | 1.80 | (-)* | |  | | | | | |
| *GFP* |  | 42 | 46.9 ± 8.4 | 1-198 |  |  |  |  | |  | | | | | |
| *LRRD7* | 80 (±11) | 72 | 93.3 ± 8.0 | 1-231 | 0.00 | 0.006 | 1.93 | (-)* | |  | | | | | |
| *GFP* |  | 45 | 66.9 ± 13.0 | 1-350 |  |  |  |  | |  | | | | | |
| *APOD* | 40 (±7) | 51 | 115.9 ± 14.8 | 1-500 | 0.54 | 0.009 | 1.67 | (-)* | |  | | | | | |
| *GFP* |  | 42 | 86.9 ± 9.1 | 1-200 |  |  |  |  | |  | | | | | |
| *IRSP1* | 78 (±9) | 63 | 120.5 ± 8.6 | 1-260 | 0.20 | 0.032 | 1.34 | (-)* | |  | | | | | |
| *GFP* |  | 42 | 87.4 ± 9.0 | 1-200 |  |  |  |  | |  | | | | | |
| *IRSP2* | 72 (±12) | 51 | 119.5 ± 10.7 | 1-350 | 0.83 | 0.159 | 1.33 | NS | |  | | | | | |
| *GFP* |  | 39 | 70.0 ± 10.0 | 1-189 |  |  |  |  | |  | | | | | |
| *FBN9* | 66 (±18) | 57 | 113.0 ± 9.8 | 1-285 | 0.34 | 0.009 | 1.56 | (-)* | |  | | | | | |
| *GFP* |  | 39 | 70.0 ± 10.0 | 1-189 |  |  |  |  | |  | | | | | |
| *FBN8* | 71 (±6) | 57 | 112.3 ± 9.9 | 1-256 | 0.05 | 0.016 | 1.55 | (-)* | |  | | | | | |
| *GFP* |  | 42 | 108.0 ± 18.9 | 1-250 |  |  |  |  | |  | | | | | |
| *GAMBICIN* | 80 (±12) | 54 | 178.7 ± 13.5 | 1-375 | 0.12 | 0.004 | 1.61 | (-)* | |  | | | | | |
| *GFP* |  | 45 | 66.8 ± 13.0 | 1-350 |  |  |  |  | |  | | | | | |
| *IRSP5* | 33 (±11) | 57 | 95.7 ± 10.5 | 1-330 | 0.53 | 0.027 | 1.38 | (-)* | |  | | | | | |
| *GFP* |  | 45 | 39.9 ± 4.9 | 1-104 |  |  |  |  | |  | | | | | |
| *GNBPB1* | 80 (±11) | 72 | 63.2 ± 8.2 | 1-252 | 0.08 | 0.037 | 1.54 | (-)* | |  | | | | | |
| *GFP* |  | 45 | 127.2 ± 21.9 | 1-420 |  |  |  |  | |  | | | | | |
| *TEP4* | 81 (±12) | 51 | 117.7 ± 12.4 | 1-370 | 0.05 | 0.418 | -1.12 | NS | |  | | | | | |
| *GFP* |  | 45 | 72.4 ± 10.8 | 1-184 |  |  |  |  | |  | | | | | |
| *PGRP-AMIDASE* | 77 (±7) | 42 | 68.4 ± 8.2 | 1-213 | 0.88 | 0.453 | -1.11 | NS | |  | | | | | |
| *GFP* |  | 42 | 54.9 ± 8.0 | 1-115 |  |  |  |  | |  | | | | | |
| *AgBP1* | 55 (±8) | 39 | 52.0 ± 6.2 | 1-153 | 0.90 | 0.646 | -1.10 | NS | |  | | | | | |
| *GFP* |  | 45 | 100.6 ± 14.7 | 1-365 |  |  |  |  | |  | | | | | |
| *HECY1* | 33 (±7) | 42 | 110.3 ± 12.4 | 1-260 | 0.75 | 0.772 | 1.03 | NS | |  | | | | | |
| *GFP* |  | 45 | 66.8 ± 13.0 | 1-350 |  |  |  |  | |  | | | | | |
| *KIN* | 27 (±6) | 66 | 72.4 ± 9.2 | 1-310 | 0.00 | 0.646 | 1.05 | NS | |  | | | | | |
| *Plasmodium* *berghei* oocyst loads in midguts of gene knockdowns (KD) and their controls (GFP). The KD and GFP mosquitoes in each dataset were fed on the same infected mouse. Data represent a pool of at least three independent randomly selected experiments with equal numbers of midguts . The efficiency of gene KD (%) on average, the total midgut numbers (midguts #), mean, and standard error of oocyst numbers (Mean±SE), range of oocyst numbers (range), *n-*fold difference of the mean oocyst numbers between gene KD and control (GFP) mosquitoes, and the p value from Kolmogorov-Smirnov test and Mann-Whitney test are presented. The repressive (-) effects of genes on parasite survival are shown in parentheses, with asterisks indicating the statistical significance at the 95% confidence level. NS indicates not significantly different. For calculation of mean oocyst numbers, midguts with zero oocysts were excluded. | | | | | | | | |  | |  |  |  |  |  |

**Table S7.** List of selected MD-2-like (ML) proteins.

| Sequence Name | accession# | Protein Name | Species |
| --- | --- | --- | --- |
| HS MD-2 | NM_006432 | MD-2 | *Homo sapiens* |
| HS MD-1 | NM_004271 | MD-1 | *Homo sapiens* |
| HS Npc2 | NM_006432 | Npc2 | *Homo sapiens* |
| Der P2 | S70378 | Der P2 | *Dermatophagoides farinae* |
| Bm PP | AB030701 | PP | *Bombyx mori* |
| Dm ML1A | CG11315 | ML1A | *Drosophila melanogaster* |
| Dm ML1B | CG11314 | ML1B | *Drosophila melanogaster* |
| Dm ML2 | CG3153 | ML2 | *Drosophila melanogaster* |
| Dm ML3 | CG7291 | ML3 | *Drosophila melanogaster* |
| Dm ML4 | CG6164 | ML4 | *Drosophila melanogaster* |
| Dm ML5A | CG3934 | ML5A | *Drosophila melanogaster* |
| Dm ML5B | CG12813 | ML5B | *Drosophila melanogaster* |
| Dm ML5C | CG31410 | ML5C | *Drosophila melanogaster* |
| AgMDL1 | [E17594](http://www.ensembl.org/Anopheles_gambiae/geneview?gene=ENSANGG00000017594) | MDL1 | *Anopheles gambiae* |
| AgMDL2 | E14474 | MDL2 | *Anopheles gambiae* |
| AgMDL3 | E14514 | MDL3 | *Anopheles gambiae* |
| AgMDL4 | E14433 | MDL4 | *Anopheles gambiae* |
| AgMDL5 | E24622 | MDL5 | *Anopheles gambiae* |
| AgMDL6 | E14418 | MDL6 | *Anopheles gambiae* |
| AgMDL7 | E14283 | MDL7 | *Anopheles gambiae* |
| AgMDL8 | E14439 | MDL8 | *Anopheles gambiae* |
| AgMDL9 | E14522 | MDL9 | *Anopheles gambiae* |
| AgMDL10 | E24912 | MDL10 | *Anopheles gambiae* |
| AgMDL11 | E16945 | MDL11 | *Anopheles gambiae* |
| AgMDL12 | E15885 | MDL12 | *Anopheles gambiae* |
| AgMDL13 | E13148 | MDL13 | *Anopheles gambiae* |
