# Supplementary material for: Anopheles gambiae Immune Responses to Human and Rodent Plasmodium Parasite Species
Source: PLoS Pathog. 2006 Jun 9;2(6):e52. doi: 10.1371/journal.ppat.0020052 (PMC1475661; doi:10.1371/journal.ppat.0020052)
Supplement: Supplementary file 2 [file ppat.0020052.sg001.doc]

**Figure S1.** Validation of microarray-assayed gene expression with real-time quantitative RT-PCR. The mean values for the expression data (log2 ratio) for 15 genes from three midgut assays (*P.f.* ookinete, *P.b.* ookinete, *P.f.* blood) obtained by microarray analysis were plotted against the corresponding mean expression values obtained with real-time RT-PCR from two biological replicates of each experiment. The Pearson correlation coefficient (p =0.86), the best-fit linear-regression analysis (R^2^ = 0.75), and the slope of the regression line (m = 0.996) demonstrated a high degree of correlation of the magnitude of regulation between the two assays. The individual values for all these genes are presented in Table S4.
