# Supplementary material for: Anopheles gambiae Immune Responses to Human and Rodent Plasmodium Parasite Species
Source: PLoS Pathog. 2006 Jun 9;2(6):e52. doi: 10.1371/journal.ppat.0020052 (PMC1475661; doi:10.1371/journal.ppat.0020052)
Supplement: Supplementary file 3 [file ppat.0020052.st003.doc]

**Table S3.** Primers used to produce PCR amplicons for dsRNA synthesis, real-time QRT-PCR for microarray validation, and verification of gene silencing.

| Primer Name | Gene ID | ENSANGT# (last 5 digits) | Primer sequence |
| --- | --- | --- | --- |
| *Tep1*-F | E14368 | 16857 | 5’- TAATACGACTCACTATAGGTTTGTGGGCCTTAAAGCGCTG -3’ |
| *Tep1*-R |  |  | 5’- TAATACGACTCACTATAGGACCACGTAACCGCTCGGTAAG-3’ |
| *Tep1-*veriF |  |  | 5’- GGTGAATCAACGGTACGTTA -3’ |
| *AgMDL1*-F | E17594 | 20083 | 5’- TAATACGACTCACTATAGGTTCGATTTTACCCCCGAGT-3’ |
| *AgMDL1*-R |  |  | 5’- TAATACGACTCACTATAGGTCCTAAGTGCGCTTGCTCT-3’ |
| *AgMDL1-*veriF |  |  | 5' - GTACAATCCATGAGGTTTCGATC - 3' |
| *LRRD7*-F | [E19333](http://www.ensembl.org/Anopheles_gambiae/geneview?gene=ENSANGG00000019333&db=core) | 21822 | 5’- TAATACGACTCACTATAGTCGGTGAGCAACAGTTTGAC-3’ |
| *LRRD7-*R |  |  | 5’- TAATACGACTCACTATAGCTTCATTCCCGCTAATGCTC-3’ |
| *LRRD7*-veriF |  |  | 5' - CGCCACGATCGAAAGCACCGCGT - 3' |
| *SPCLIP1*-F | [E17669](http://www.ensembl.org/Anopheles_gambiae/geneview?gene=ENSANGG00000017669&db=core) | 20158 | 5’- TAATACGACTCACTATAGGTCACCGAACACGTCCAAC-3’ |
| *SPCLIP1*-R |  |  | 5’- TAATACGACTCACTATAGCTGCATGGCCCTACGTCTA-3’ |
| *SPCLIP-*veriF |  |  | 5' - GCCGTAGAGGAGCTTATCATCCA - 3' |
| *IRSP2*-F | [E1673](http://www.ensembl.org/Anopheles_gambiae/geneview?gene=ENSANGG00000016731&db=core)1 | 19220 | 5’- TAATACGACTCACTATAGGCTAGCGTTGTCGTTTGCTC-3’ |
| *IRSP2*-R |  |  | 5’- TAATACGACTCACTATAGTGACATTTCAATCCCATTCG-3’ |
| *IRSP2*-veriF |  |  | 5’- CTAATGCGCTCGGTGCGGGCAAT -3’ |
| *IRSP1*-F | [E18539](http://www.ensembl.org/Anopheles_gambiae/geneview?gene=ENSANGG00000018539&db=core) | 21028 | 5’-TAATACGACTCACTATAGCTGGAAGGATGGACAGATGG-3’ |
| *IRSP1*-R |  |  | 5’- TAATACGACTCACTATAGGGATTGAAACGGATCAAGG-3’ |
| *IRSP1-*veriF |  |  | 5' - CCGATCGGACACTTCACCCAGAT - 3' |
| *IRSP5*-F | E16962 | 19451 | 5’- TAATACGACTCACTATAGCAGTTTGCTGTCTGCTGCTC |
| *IRSP5*-R |  |  | 5’- TAATACGACTCACTATAGTCGCTCTCAATCTTCCAGTG |
| *IRSP5-*veriF |  |  | 5' - GATATCGATTGCTGGTACTGTTG - 3' |
| *PGRP-AMIDASE*-F | E10489 | 12978 | 5’- TAATACGACTCACTATAGAGGATAGGCGGCAGTGAAC-3’ |
| *PGRP-AMIDASE* -R |  |  | 5’- TAATACGACTCACTATAGTCCATGTGGAAGGACTGGA-3’ |
| *PGRP-AMIDASE -*veriF |  |  | 5’- CGATAAGTAATCGACAATTCT - 3' |
| *Gambicin*-F | E10766 | 13255 | 5’- TAATACGACTCACTATAGGGCTATCTCAACCGGAAGG-3’ |
| *Gambicin*-R |  |  | 5’- TAATACGACTCACTATAGCCAACGTCTGGCACTGATT-3’ |
| *Gambicin-*veriF |  |  | 5' - CGGCAGCCGTTGCGGATGCAATG - 3' |
| *Tep4*-F | [E18727](http://www.ensembl.org/Anopheles_gambiae/geneview?gene=ENSANGG00000018727&db=core) | 21216 | 5’- TAATACGACTCACTATAGTGTTGATTGCACTGCTGGA-3’ |
| *Tep4*-R |  |  | 5’- TAATACGACTCACTATAGGAGGTCCCGTCCACGTACT -3’ |
| *Tep4-*veriF |  |  | 5' - GGAGGGGCCGTCTTTCTGACCGC - 3' |
| *AgBP1*-F | [E05241](http://www.ensembl.org/Anopheles_gambiae/geneview?gene=ENSANGG00000005241&db=core) | 06959 | 5’- TAATACGACTCACTATAGCATTCCCCTGCTGAAGGATA-3’ |
| *AgBP1*-R |  |  | 5’- TAATACGACTCACTATAGATCTTGCGCAACGGATACTC-3’ |
| *AgBP-*veriF |  |  | 5’- GTTCACCGGTGACAGCGTGCTC -3’ |
| *APOD*-F | E16962 | 28106 | 5’- TAATACGACTCACTATAGTAGCTATCCGGACGTGTTCC-3’ |
| *APOD*-R |  |  | 5’- TAATACGACTCACTATAGTTGCATTTTGCCTGACTTTG-3’ |
| *APOD-*veriF |  |  | 5' - CGAGAAGGACTGTGACTGCGGGT - 3' |
| *HECY1*-F | [E19569](http://www.ensembl.org/Anopheles_gambiae/geneview?gene=ENSANGG00000019569&db=core) | 22058 | 5’- TAATACGACTCACTATAGTCGAACGATCTCGGTACGATT -3’ |
| *HECY1*-R |  |  | 5’- TAATACGACTCACTATAGTCCGTTCGATTGGATCAGATT -3’ |
| *HECY1-*veriF |  |  | 5' - GTGCGTTCATCCCGAGCAACTAC - 3' |
| *KIN*-F | E11642 | 14131 | 5’- TAATACGACTCACTATAGTTTGTTGGCGATCTTTGTCA -3’ |
| *KIN*-R |  |  | 5’- TAATACGACTCACTATAGGCATGATCACATTTCCCCTAGT -3’ |
| *KIN-*veriF |  |  | 5' - CTGACCAGTCCGCCCGGAGACGC - 3' |
| *FBN9-F* | E08759 | 11248 | 5’- TAATACGACTCACTATAGCCAAGATGTCGGGCAAGTAT -3’ |
| *FBN9-R* |  |  | 5’- TAATACGACTCACTATAGTTGTGGTACGTCAGCGAGTC -3’ |
| *FBN9-*veriF |  |  | 5’- CGCTCCCTGTTCGAGCTGCA -3’ |
| *FBN8-F* | E08763 | 11252 | 5’- TAATACGACTCACTATAGTTCAAGGGCAATTTTTGGTC -3’ |
| *FBN8-R* |  |  | 5’- TAATACGACTCACTATAGATATTGTTCCGCCTCACTGC -3’ |
| *FBN8-*veriF |  |  | 5’- CCACGAACAAATGCGGAACG -3’ |
| *FBN39-F* | E18891 | 21380 | 5’- TAATACGACTCACTATAGCGCTACACCCTGTTCGAGAT -3’ |
| *FBN39-R* |  |  | 5’- TAATACGACTCACTATAGCCGTACTGGTCGTGGTCCTG -3’ |
| *FBN39-*veriF |  |  | 5’-GGCCGAATCGTTCAAGGGCG -3’ |
| *GNBPB1-F* | E15205 | 17694 | 5’- TAATACGACTCACTATAGGTTCGGGTTCGTGTGGAC -3’ |
| *GNBPB1-R* |  |  | 5’- TAATACGACTCACTATAG ACCAGCAACGAGGCAGAC -3’ |
| *GNBPB1-*veriF |  |  | 5’- ACGGCAACGCTAACGAAG -3’ |
| *IRSP3-F* | [E22382](http://www.ensembl.org/Anopheles_gambiae/geneview?gene=ENSANGG00000022382&db=core) | 09630 | 5’- TAATACGACTCACTATAGAGTCGTTCAACCAGGGCTTC -3’ |
| *IRSP3-R* |  |  | 5’- TAATACGACTCACTATAGCCAGTTTCGAGGCAGAGGTA -3’ |
| Underlined letters indicated the T7 promoter sequence. The same pair of forward and reverse primers was used for both dsRNA synthesis and QT-RT PCR validation of microarray expression data. For the RT-PCR verification of gene silencing, the different veriF primers and reverse primers were used. | | | |
