# Supplementary material for: Anopheles gambiae Immune Responses to Human and Rodent Plasmodium Parasite Species
Source: PLoS Pathog. 2006 Jun 9;2(6):e52. doi: 10.1371/journal.ppat.0020052 (PMC1475661; doi:10.1371/journal.ppat.0020052)
Supplement: Supplementary file 4 [file ppat.0020052.st004.doc]

**Table S4.** Correlation of microarray expression data with real-time QRT-PCR

|  | ***P.f.* ookinete (gut)** | |  | ***P.b.* ookinete (gut)** | |  | ***P.f.* blood (gut)** | |  |  |
| --- | --- | --- | --- | --- | --- | --- | --- | --- | --- | --- |
|  | QRT ± SE | Arrays ± SE |  | QRT ± SE | Arrays ± SE |  | QRT ± SE | Arrays ± SE |  | P |
| *Tep1* | 2.30 ± 0.14 | 2.26 ± 0.09 |  | 0.82 ± 0.09 | -0.30 ± 0.01 |  | -0.28 ± 0.09 | 0.14 ± 0.10 |  | 0.83 |
| *AgMDL1* | 1.43 ± 0.02 | 1.57 ± 0.21 |  | -1.08 ± 0.08 | -0. 09 ± 0.23 | | -0.44 ± 0.11 | -0.87 ± 0.08 |  | 0.84 |
| *SPCLIP1* | 1.51 ± 0.02 | 1.80 ± 0.07 |  | -0.33 ± 0.06 | -0.32 ± 0.07 |  | -0.33 ± 0.13 | -0.06 ± 0.06 |  | 0.99 |
| *LRRD7* | 2.23 ± 0.12 | 2.18 ± 0.25 |  | -1.87 ± 0.03 | -1.62 ± 0.15 |  | -1.56 ± 0.10 | 1.02 ± 0.11 |  | 0.78 |
| *APOD* | 0.91 ± 0.08 | 1.30 ± 0.12 |  | -1.99 ± 0.10 | -0.89 ± 0.03 |  | 0.43 ± 0.19 | -0.07 ± 0.01 |  | 0.87 |
| *IRSP1* | 2.34 ± 0.22 | 2.33 ± 0.35 |  | -0.7 ± 0.18 | -0.06 ± 0.12 |  | N/A | N/A |  | N/A |
| *IRSP2* | 2.00 ± 0.05 | 2.24 ± 0.23 |  | -0.99 ± 0.12 | -0.44 ± 0.03 |  | -2.69 ± 0.09 | -2.20 ± 0.26 |  | 0.99 |
| *GAMBICIN* | 0.02 ± 0.26 | -0.30 ± 0.05 | | 0.24 ± 0.11 | 0.06 ± 0.26 |  | 3.53 ± 0.20 | 3.15 ± 0.36 |  | 0.90 |
| *IRSP5* | 2.45 ± 0.05 | 2.90 ± 0.26 |  | -2.14 ± 0.04 | -2.10 ± 0.10 |  | N/A | N/A |  | N/A |
| *TEP4* | 2.41 ± 0.06 | 1.66 ± 0.12 |  | 0.56 ± 0.02 | -0.17 ± 0.10 |  | -0.026 ± 0.05 | 0.03 ± 0.02 |  | 0.89 |
| *KIN* | 1.45 ± 0.11 | 1.77 ± 0.13 |  | -1.61 ± 0.10 | -0.49 ± 0.12 |  | 2.16 ± 0.02 | 1.16 ± 0.03 |  | 0.90 |
| *PGRP-AMIDASE* | 0.90 ± 0.10 | -0.02 ± 0.14 | | 1.03 ± 0.13 | 1.64 ± 0.28 |  | 2.15 ± 0.28 | 1.40 ± 0.09 |  | 0.93 |
| *AgBP1* | 1.87 ± 0.17 | 1.78 ± 0.17 |  | -0.13 ± 0.03 | -0.99 ± 0.01 |  | -0.48 ± 0.13 | -0.76 ± 0.09 |  | 0.98 |
| *HECY1* | 2.32 ± 0.07 | 1.54 ± 0.15 |  | -3.57 ± 0.23 | N/A |  | -0.96 ± 0.20 | -0.73 ± 0.06 |  | N/A |
| *IRSP6* | 1.97 ± 0.05 | 2.31 ± 0.06 |  | -0.13 ± 0.04 | 0.02 ± 0.05 |  | 3.23 ± 0.05 | 1.36 ± 0.16 |  | 0.79 |

Comparison of the expression data from real-time quantitative RT-PCR (QRT) and DNA microarrays (Arrays) for 15 genes. For QRT-PCR, data were obtained from two biological and three technical replicates. The mean value for the regulation and standard error of the mean (SE) for the reactions were obtained from both QRT-PCR and array data. Pearson correlation (P) indicated the consistency between the two methods. N/A indicates the absence of microarray data.
