# Supplementary material for: Anopheles gambiae Immune Responses to Human and Rodent Plasmodium Parasite Species
Source: PLoS Pathog. 2006 Jun 9;2(6):e52. doi: 10.1371/journal.ppat.0020052 (PMC1475661; doi:10.1371/journal.ppat.0020052)
Supplement: Supplementary file 5 [file ppat.0020052.st005.doc]

**Table S5.** Effect of gene silencing on *P. falciparum* infection (oocyst numbers)

| Gene | Midguts # | Mean Oocysts ± SE | Oocysts(range) | p value KSTest | p value Mann-Whitney | *n*-Fold Difference | Significance |  | | | | | |
| --- | --- | --- | --- | --- | --- | --- | --- | --- | --- | --- | --- | --- | --- |
| *GFP* | 54 | 1.12 ± 0.36 | 0-6 | 0.00 |  |  |  |  | | | | | |
| *Tep1* | 48 | 2.32 ± 0.49 | 0-14 | 0.01 | 0.026 | 2.06 | (-)* |  | | | | | |
| *GFP* | 48 | 1.96 ± 0.24 | 0-9 |  |  |  |  |  | | | | | |
| *AgMDL1* | 75 | 4.06 ± 0.61 | 0-22 | 0.05 | 0.005 | 2.07 | (-)* |  | | | | | |
| *GFP* | 51 | 1.28 ± 0.22 | 0-5 |  |  |  |  |  | | | | | |
| *FBN39* | 63 | 2.02 ± 0.37 | 0-7 | 0.01 | 0.049 | 1.58 | (-)* |  | | | | | |
| *GFP* | 75 | 1.13 ± 0.19 | 0-6 |  |  |  |  |  | | | | | |
| *SPCLIP1* | 93 | 1.98 ± 0.36 | 0-12 | 0.00 | 0.011 | 1.76 | (-)* |  | | | | | |
| *GFP* | 84 | 1.26 ± 0.17 | 0-6 |  |  |  |  |  | | | | | |
| *LRRD7* | 114 | 2.24 ± 0.49 | 0-27 | 0.03 | 0.010 | 1.78 | (-)* |  | | | | | |
| *GFP* | 39 | 4.70 ± 0.46 | 0-9 |  |  |  |  |  | | | | | |
| *APOD* | 63 | 11.00 ± 0.60 | 0-63 | 0.00 | 0.003 | 2.34 | (-)* |  | | | | | |
| *GFP* | 45 | 2.36 ± 0.43 | 0-9 |  |  |  |  |  | | | | | |
| *IRSP1* | 54 | 4.88 ± 0.62 | 0-51 | 0.01 | 0.045 | 2.07 | (-)* |  | | | | | |
| *GFP* | 63 | 1.26 ± 0.36 | 0-6 |  |  |  |  |  | | | | | |
| *IRSP2* | 72 | 1.77 ± 0.37 | 0-24 | 0.01 | 0.233 | 1.41 | NS |  | | | | | |
| *GFP* | 51 | 1.28 ± 0.42 | 0-5 |  |  |  |  |  | | | | | |
| *FBN9* | 60 | 2.18 ± 0.32 | 0-6 | 0.02 | 0.018 | 1.70 | (-)* |  | | | | | |
| *GFP* | 51 | 1.28 ± 0.20 | 0-5 |  |  |  |  |  | | | | | |
| *FBN8* | 63 | 2.02 ± 0.32 | 0-7 | 0.01 | 0.045 | 1.58 | (-)* |  | | | | | |
| *GFP* | 45 | 2.36 ± 0.21 | 0-9 |  |  |  |  |  | | | | | |
| *GAMBICIN* | 66 | 2.67 ± 0.38 | 0-20 | 0.01 | 0.245 | 1.13 | NS |  | | | | | |
| *GFP* | 45 | 2.36 ± 0.21 | 0-9 |  |  |  |  |  | | | | | |
| *IRSP5* | 54 | 2.07 ± 0.39 | 0-29 | 0.01 | 0.356 | -1.14 | NS |  | | | | | |
| *Plasmodium* *falciparum* oocyst loads in midguts of gene knockdowns (KD) and their controls (GFP). The efficiency of gene KD (%) is presented in Table S6. The KD and GFP control mosquitoes in each dataset were fed on the same *P. falciparum* gametocyte culture. The results of equal numbers of midguts from all three experiments in each dataset were pooled. The total midgut numbers (midguts #), mean and standard error of oocyst numbers (Mean±SE), range of oocyst numbers (range), *n-*fold difference of the mean oocyst numbers between gene KD and control (GFP) mosquitoes, and the p-value from two independent probability tests (Kolmogorov-Smirnov (KS) and Mann-Whitney test*)* are presented. Zero oocysts are also included for calculation of mean oocyst numbers. The repressive (-) effects of genes on parasite survival are shown in parentheses, with the asterisks indicating statistical significant at the 95% confidence level. NS indicates not significantly different. For calculation of mean oocyst numbers, midguts with zero oocysts were included. | | | | | | | | |  |  |  |  |  |
