# Supplementary material for: Anopheles gambiae Immune Responses to Human and Rodent Plasmodium Parasite Species
Source: PLoS Pathog. 2006 Jun 9;2(6):e52. doi: 10.1371/journal.ppat.0020052 (PMC1475661; doi:10.1371/journal.ppat.0020052)
Supplement: Supplementary file 6 [file ppat.0020052.st006.doc]

**Table S6.** Effect of gene silencing on *P. berghei* infection (oocyst numbers)

| Gene | KD% (± SE) | Midguts # | Mean Oocysts ± SE | Oocysts(range) | p value KS-Test | p value Mann-Whitney | *n*-Fold Difference | Signif icance | |  | | | | | |
| --- | --- | --- | --- | --- | --- | --- | --- | --- | --- | --- | --- | --- | --- | --- | --- |
| *GFP* |  | 42 | 93.0 ± 12.8 | 1-296 | 0.00 |  | 1.00 |  | |  | | | | | |
| *Tep1* | 78 (±9) | 42 | 205.6 ± 21.7 | 1-500 | 0.00 | 0.000 | 2.21 | (-)* | |  | | | | | |
| *GFP* |  | 45 | 87.2 ± 8.3 | 1-198 |  |  |  |  | |  | | | | | |
| *AgMDL1* | 66 (±8) | 78 | 60.2 ± 4.8 | 1-185 | 0.01 | 0.051 | -1.49 | NS | |  | | | | | |
| *GFP* |  | 39 | 70.0 ± 10.0 | 1-189 |  |  |  |  | |  | | | | | |
| *FBN39* | 58 (±10) | 57 | 84.8 ± 7.6 | 1-213 | 0.03 | 0.374 | 1.17 | NS | |  | | | | | |
| *GFP* |  | 36 | 82.0 ± 14.7 | 1-320 |  |  |  |  | |  | | | | | |
| *SPCLIP1* | 88 (±8) | 48 | 152.7 ± 14.0 | 1-430 | 0.00 | 0.001 | 1.80 | (-)* | |  | | | | | |
| *GFP* |  | 42 | 46.9 ± 8.4 | 1-198 |  |  |  |  | |  | | | | | |
| *LRRD7* | 80 (±11) | 72 | 93.3 ± 8.0 | 1-231 | 0.00 | 0.006 | 1.93 | (-)* | |  | | | | | |
| *GFP* |  | 45 | 66.9 ± 13.0 | 1-350 |  |  |  |  | |  | | | | | |
| *APOD* | 40 (±7) | 51 | 115.9 ± 14.8 | 1-500 | 0.54 | 0.009 | 1.67 | (-)* | |  | | | | | |
| *GFP* |  | 42 | 86.9 ± 9.1 | 1-200 |  |  |  |  | |  | | | | | |
| *IRSP1* | 78 (±9) | 63 | 120.5 ± 8.6 | 1-260 | 0.20 | 0.032 | 1.34 | (-)* | |  | | | | | |
| *GFP* |  | 42 | 87.4 ± 9.0 | 1-200 |  |  |  |  | |  | | | | | |
| *IRSP2* | 72 (±12) | 51 | 119.5 ± 10.7 | 1-350 | 0.83 | 0.159 | 1.33 | NS | |  | | | | | |
| *GFP* |  | 39 | 70.0 ± 10.0 | 1-189 |  |  |  |  | |  | | | | | |
| *FBN9* | 66 (±18) | 57 | 113.0 ± 9.8 | 1-285 | 0.34 | 0.009 | 1.56 | (-)* | |  | | | | | |
| *GFP* |  | 39 | 70.0 ± 10.0 | 1-189 |  |  |  |  | |  | | | | | |
| *FBN8* | 71 (±6) | 57 | 112.3 ± 9.9 | 1-256 | 0.05 | 0.016 | 1.55 | (-)* | |  | | | | | |
| *GFP* |  | 42 | 108.0 ± 18.9 | 1-250 |  |  |  |  | |  | | | | | |
| *GAMBICIN* | 80 (±12) | 54 | 178.7 ± 13.5 | 1-375 | 0.12 | 0.004 | 1.61 | (-)* | |  | | | | | |
| *GFP* |  | 45 | 66.8 ± 13.0 | 1-350 |  |  |  |  | |  | | | | | |
| *IRSP5* | 33 (±11) | 57 | 95.7 ± 10.5 | 1-330 | 0.53 | 0.027 | 1.38 | (-)* | |  | | | | | |
| *GFP* |  | 45 | 39.9 ± 4.9 | 1-104 |  |  |  |  | |  | | | | | |
| *GNBPB1* | 80 (±11) | 72 | 63.2 ± 8.2 | 1-252 | 0.08 | 0.037 | 1.54 | (-)* | |  | | | | | |
| *GFP* |  | 45 | 127.2 ± 21.9 | 1-420 |  |  |  |  | |  | | | | | |
| *TEP4* | 81 (±12) | 51 | 117.7 ± 12.4 | 1-370 | 0.05 | 0.418 | -1.12 | NS | |  | | | | | |
| *GFP* |  | 45 | 72.4 ± 10.8 | 1-184 |  |  |  |  | |  | | | | | |
| *PGRP-AMIDASE* | 77 (±7) | 42 | 68.4 ± 8.2 | 1-213 | 0.88 | 0.453 | -1.11 | NS | |  | | | | | |
| *GFP* |  | 42 | 54.9 ± 8.0 | 1-115 |  |  |  |  | |  | | | | | |
| *AgBP1* | 55 (±8) | 39 | 52.0 ± 6.2 | 1-153 | 0.90 | 0.646 | -1.10 | NS | |  | | | | | |
| *GFP* |  | 45 | 100.6 ± 14.7 | 1-365 |  |  |  |  | |  | | | | | |
| *HECY1* | 33 (±7) | 42 | 110.3 ± 12.4 | 1-260 | 0.75 | 0.772 | 1.03 | NS | |  | | | | | |
| *GFP* |  | 45 | 66.8 ± 13.0 | 1-350 |  |  |  |  | |  | | | | | |
| *KIN* | 27 (±6) | 66 | 72.4 ± 9.2 | 1-310 | 0.00 | 0.646 | 1.05 | NS | |  | | | | | |
| *Plasmodium* *berghei* oocyst loads in midguts of gene knockdowns (KD) and their controls (GFP). The KD and GFP mosquitoes in each dataset were fed on the same infected mouse. Data represent a pool of at least three independent randomly selected experiments with equal numbers of midguts . The efficiency of gene KD (%) on average, the total midgut numbers (midguts #), mean, and standard error of oocyst numbers (Mean±SE), range of oocyst numbers (range), *n-*fold difference of the mean oocyst numbers between gene KD and control (GFP) mosquitoes, and the p value from Kolmogorov-Smirnov test and Mann-Whitney test are presented. The repressive (-) effects of genes on parasite survival are shown in parentheses, with asterisks indicating the statistical significance at the 95% confidence level. NS indicates not significantly different. For calculation of mean oocyst numbers, midguts with zero oocysts were excluded. | | | | | | | | |  | |  |  |  |  |  |
