# Supplementary material for: Anopheles gambiae Immune Responses to Human and Rodent Plasmodium Parasite Species
Source: PLoS Pathog. 2006 Jun 9;2(6):e52. doi: 10.1371/journal.ppat.0020052 (PMC1475661; doi:10.1371/journal.ppat.0020052)
Supplement: Supplementary file 7 [file ppat.0020052.st007.doc]

**Table S7.** List of selected MD-2-like (ML) proteins.

| Sequence Name | accession# | Protein Name | Species |
| --- | --- | --- | --- |
| HS MD-2 | NM_006432 | MD-2 | *Homo sapiens* |
| HS MD-1 | NM_004271 | MD-1 | *Homo sapiens* |
| HS Npc2 | NM_006432 | Npc2 | *Homo sapiens* |
| Der P2 | S70378 | Der P2 | *Dermatophagoides farinae* |
| Bm PP | AB030701 | PP | *Bombyx mori* |
| Dm ML1A | CG11315 | ML1A | *Drosophila melanogaster* |
| Dm ML1B | CG11314 | ML1B | *Drosophila melanogaster* |
| Dm ML2 | CG3153 | ML2 | *Drosophila melanogaster* |
| Dm ML3 | CG7291 | ML3 | *Drosophila melanogaster* |
| Dm ML4 | CG6164 | ML4 | *Drosophila melanogaster* |
| Dm ML5A | CG3934 | ML5A | *Drosophila melanogaster* |
| Dm ML5B | CG12813 | ML5B | *Drosophila melanogaster* |
| Dm ML5C | CG31410 | ML5C | *Drosophila melanogaster* |
| AgMDL1 | [E17594](http://www.ensembl.org/Anopheles_gambiae/geneview?gene=ENSANGG00000017594) | MDL1 | *Anopheles gambiae* |
| AgMDL2 | E14474 | MDL2 | *Anopheles gambiae* |
| AgMDL3 | E14514 | MDL3 | *Anopheles gambiae* |
| AgMDL4 | E14433 | MDL4 | *Anopheles gambiae* |
| AgMDL5 | E24622 | MDL5 | *Anopheles gambiae* |
| AgMDL6 | E14418 | MDL6 | *Anopheles gambiae* |
| AgMDL7 | E14283 | MDL7 | *Anopheles gambiae* |
| AgMDL8 | E14439 | MDL8 | *Anopheles gambiae* |
| AgMDL9 | E14522 | MDL9 | *Anopheles gambiae* |
| AgMDL10 | E24912 | MDL10 | *Anopheles gambiae* |
| AgMDL11 | E16945 | MDL11 | *Anopheles gambiae* |
| AgMDL12 | E15885 | MDL12 | *Anopheles gambiae* |
| AgMDL13 | E13148 | MDL13 | *Anopheles gambiae* |
